# Single‐cell RNA sequencing captures patient‐level heterogeneity and associated molecular phenotypes in breast cancer pleural effusions

**DOI:** 10.1002/ctm2.1356

**Published:** 2023-09-10

**Authors:** Holly J. Whitfield, Jean Berthelet, Stefano Mangiola, Caroline Bell, Robin L. Anderson, Bhupinder Pal, Belinda Yeo, Anthony T. Papenfuss, Delphine Merino, Melissa J. Davis

**Affiliations:** ^1^ Department of Medical Biology, The Faculty of Medicine Dentistry and Health Science, The University of Melbourne Carlton Victoria Australia; ^2^ Bioinformatics Division The Walter and Eliza Hall Institute of Medical Research Parkville Victoria Australia; ^3^ Olivia Newton‐John Cancer Research Institute Heidelberg Victoria Australia; ^4^ School of Cancer Medicine La Trobe University Bundoora Victoria Australia; ^5^ Peter MacCallum Cancer Centre Parkville Victoria Australia; ^6^ Department of Clinical Pathology, Faculty of Medicine Dentistry and Health Science, The University of Melbourne Carlton Victoria Australia; ^7^ Austin Health Heidelberg Victoria Australia; ^8^ Sir Peter MacCallum Department of Oncology The University of Melbourne Carlton Victoria Australia; ^9^ Immunology Division The Walter and Eliza Hall Institute of Medical Research Parkville Victoria Australia; ^10^ The University of Queensland Diamantina Institute The University of Queensland Brisbane Queensland Australia; ^11^ The South Australian Immunogenomics Cancer Institute The University of Adelaide Adelaide South Australia Australia

## Abstract

**Background:**

Malignant pleural effusions (MPEs) are a common complication of advanced cancers, particularly those adjacent to the pleura, such as lung and breast cancer. The pathophysiology of MPE formation remains poorly understood, and although MPEs are routinely used for the diagnosis of breast cancer patients, their composition and biology are poorly understood. It is difficult to distinguish invading malignant cells from resident mesothelial cells and to identify the directionality of interactions between these populations in the pleura. There is a need to characterize the phenotypic diversity of breast cancer cell populations in the pleural microenvironment, and investigate how this varies across patients.

**Methods:**

Here, we used single‐cell RNA‐sequencing to study the heterogeneity of 10 MPEs from seven metastatic breast cancer patients, including three Miltenyi‐enriched samples using a negative selection approach. This dataset of almost 65 000 cells was analysed using integrative approaches to compare heterogeneous cell populations and phenotypes.

**Results:**

We identified substantial inter‐patient heterogeneity in the composition of cell types (including malignant, mesothelial and immune cell populations), in expression of subtype‐specific gene signatures and in copy number aberration patterns, that captured variability across breast cancer cell populations. Within individual MPEs, we distinguished mesothelial cell populations from malignant cells using key markers, the presence of breast cancer subtype expression patterns and copy number aberration patterns. We also identified pleural mesothelial cells expressing a cancer‐associated fibroblast‐like transcriptomic program that may support cancer growth.

**Conclusions:**

Our dataset presents the first unbiased assessment of breast cancer‐associated MPEs at a single cell resolution, providing the community with a valuable resource for the study of MPEs. Our work highlights the molecular and cellular diversity captured in MPEs and motivates the potential use of these clinically relevant biopsies in the development of targeted therapeutics for patients with advanced breast cancer.

## Graphical abstract

Malignant pleural effusions (MPEs) are common complications of advanced breast cancer. However, their cellular composition is still unclear. Here we generated a novel single‐cell dataset to explore the heterogeneity of these valuable clinical samples, including cancer cells and normal cells from the tumour microenvironment.

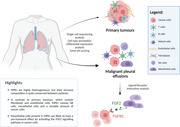

## 1 BACKGROUND

Malignant pleural effusions (MPEs) regularly develop in advanced cancers,^1^ where fluid accumulation in the pleura indicates cancer dissemination, and is associated with chest pain, dyspnoea, impaired quality of life^1^, ^2^ and poor prognosis.^3^, ^4^ With a rising global cancer incidence and improved life expectancy for breast cancer patients, an increase in MPE presentations and associated burden is expected.^1^ This includes painful treatment side effects^5^ and an augmentation in the healthcare costs associated with procedures and hospitalization,^1^ particularly for symptomatic recurrent MPEs. Treatment remains primarily palliative,^5^ involving surgical drainage or mechanical approaches to prevent recurrence,^1^, ^6^ and minimal progress has been made in the development of new, safer treatments that prevent recurrence.^6^, ^7^

Understanding the mechanisms that lead to MPE formation in breast cancer patients is crucial to the development of targeted therapeutic approaches. Breast cancer‐associated MPEs are understudied despite being the second leading cause of MPE.^7^ It is also likely that the pathogenesis, clinical presentation and recommended treatment of MPE in breast cancer patients is different from other more well‐studied cancers, such as lung and mesothelioma‐associated MPEs.^1^, ^4^, ^7^ The presence of malignant cells in the effusion implies metastasis to the pleural wall, which is thought to occur either by direct dissemination from adjacent tissue or by lymphatic invasion.^1^, ^4^, ^8^ However, up to half of breast cancer‐associated MPEs are cytology negative,^9^ suggesting a para‐malignant pleural effusion and no pleural metastasis,^1^ where the mechanism of pleural effusion formation and hence prognosis of these patients is unknown.^1^, ^8^

Our ability to diagnose pleural metastases and detect malignant cells in the pleural fluid can be challenging. While cytology and immuno‐histochemistry are useful for rapid, non‐invasive diagnosis of pleural lining metastases,^4^, ^5^ they suffer from low sensitivity and are confounded by reactive mesothelial cells that can shed into the pleural fluid.^10^, ^11^ Hence, establishing the source of cancer cells and distinguishing them from mesothelial cells has been a key area of research that has focused on establishing robust histologically validated markers.^10^, ^12^ These markers, however, are expressed heterogeneously and are further complicated by the potential involvement of mesothelial cells in cancer progression.

Many studies suggest a role for mesothelial cell‐derived cancer‐associated fibroblasts (CAFs) in the adhesion and growth of tumours along the pleural wall.^13^, ^14^ Co‐culturing assays and other experiments provide evidence for interactions between mesothelial cells and invading cancer cells, but the extent of mesothelial involvement in breast cancer metastases and the nature of these interactions is not well understood.^15^ Cancer cells are thought to secrete growth factors and promote a mesothelial‐to‐mesenchymal transition (MMT) in mesothelial cells, contributing fibroblasts to the tumour microenvironment and exposing the basement membrane to cancer cells for attachment.^16^, ^17^ Mesothelial cells can also undergo MMT in response to inflammation, suggesting that the secretion of factors by cancer cells is not necessarily required for this transformation.^18^ Some studies show that these mesothelial cell‐derived CAFs promote cancer survival through extracellular matrix (ECM) support and adequate vascularization,^13^, ^14^, ^16^ while others suggest that mesothelial cells are protective against cancer invasion.^19^, ^20^

The recent maturation of single‐cell RNA sequencing enables the phenotypic characterization of individual cells from MPEs, such as recent studies on lung cancer‐associated MPEs.^21^, ^22^ However, these studies used tumour cell enrichment approaches to focus on cancer cells. In order to fully characterized the cellular environment of breast cancer MPEs, we generated a single‐cell RNA sequencing dataset of MPEs with used an unbiased approach, in the absence of fluorescence‐activated cell sorting (FACS) or tumour‐cell enrichment strategies. This enabled us to isolate and capture these sometimes rare cells in single‐cell MPE experiments and increase our ability to systematically characterize their presence and impact in metastatic disease. We used this novel dataset to investigate interactions between mesothelial and cancer cells at single‐cell resolution, and identified how mesothelial‐like cells may be involved in pleural metastasis and MPE formation in breast cancer. This deep profiling of the cellular composition of pleural fluid will improve our understanding of MPEs in breast cancer patients.

## 2 METHODS

### 2.1 Patient sample collection

MPEs were collected in eligible breast cancer patients following written informed consent. MPEs were drained through a pleural tap (thoracentesis) or a chest tube (tube thoracostomy). In cases of recurrent pleural effusions, video‐assisted thoracoscopic surgery was performed where fluid was removed in addition to therapeutic intervention. A part of the fluid was set aside for diagnostic purposes and the remaining fluid was collected. Any pieces of tissue were removed and the fluid was centrifuged at 300 g for 7 min. MPEs showing a high degree of viscosity were diluted with phosphate buffered saline (PBS) solution to obtain a cell pellet. If the MPEs contained blood, red blood cell lysis was performed on the pellets for up to two rounds of 5 min. Cells were then washed in PBS before manual counting.

### 2.2 Sample preparation for scRNA‐seq

*S*ample processing for single‐cell capture was adapted depending on the MPE. If the sample contained a lot of aggregates, cell clumps were mechanically dissociated using a 200 μL pipette. The cell suspension was enriched for living cells using the dead cell removal kit (#130‐090‐101; Miltenyi Biotec) following the manufacturer recommendations. To enrich for human cancer cells, tumour‐associated cells, such as lymphocytes, fibroblasts and endothelial cells, were magnetically depleted using the tumour cell isolation kit, human (#130‐108‐339; Miltenyi Biotec) following the manufacturer recommendations. Magnetic depletions were done using LS columns (#130‐042‐401; Miltenyi Biotec) and the MidiMACS separator (#130‐042‐302; Miltenyi Biotec).

The single‐cell capture was done using a Chromium controller machine (#120223; 10X‐Genomics) and a Chromium single‐cell gel bead kit (#1000075; 10X‐Genomics). A total of 10 000 cells were captured in Gel Bead in Emulsions. The cDNA was then isolated and the library prepared as recommended by the manufacturer using either the 3′ V3 kit (#1000078; 10X‐Genomics) for newer samples (BCB‐0066, BCB‐0112, BCB‐0139, BCB‐0020, BCB‐0021, BCB‐0114 and BCB‐0090) or the 3′ V2 kit (#120237; 10X‐Genomics) for older samples (BCB‐0066_E, BCB‐0020_E and BCB‐0021_E). Sequencing runs were completed on a NextSeq machine (Illumina) with the following parameters: Read1 28 cycles, Read2 91 cycles and Index Read 8 cycles.

### 2.3 Data processing for visualizations

Gene counts were processed using R packages scater and scran, which included log‐normalization, TPM calculation, doublet detection and dimension reduction, following standard workflows. Cell‐type annotations were refined to ensure that only confidently assigned cells were used in downstream analyses. For analysis of non‐immune populations (Figures 3 and 4), we required that malignant cells expressed EpCAM and mesothelial cells expressed Desmin and not EpCAM. If an epithelial or mesothelial cell was negative for EpCAM or Desmin, it needed to express at least two relevant markers instead. For more details on scRNA‐seq data processing, see Supplementary Methods.

The perplexity tSNE parameter set to 20 for all figures to emphasize local differences between cells. To address batch effects that were introduced by different sequencing versions between enriched and non‐enriched samples, we used Seurat to integrate these samples for tSNE visualizations in Figure 3. To classify T cell subpopulations, ProjecTILs^24^ was applied on default settings using the T cell reference atlas provided by the package. Discordant cells were removed based on CD8A/B and CD4 expression; CD4‐positive T cells predicted to be CD8‐subtypes (CD8 Naïve‐like, CD8 Early Active, CCD8 Effector Memory) and CD8‐positive cells predicted to be CD4‐subtypes (Th1, Tfh, CD4 Naïve‐like) were excluded (Figure 2E).

**FIGURE 1:**
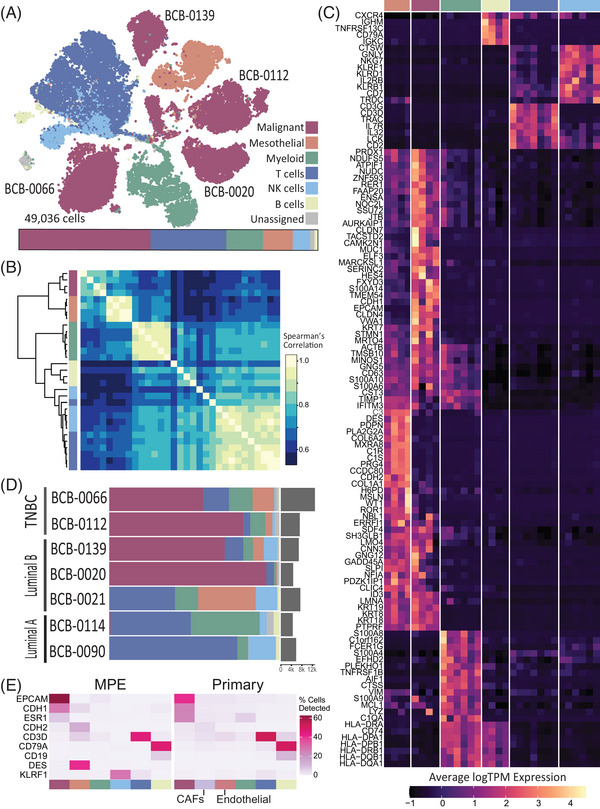
Single‐cell transcriptomic landscape of malignant pleural effusions. (A) A tSNE representation of 49 109 cells that were isolated from seven pleural effusions. Six cell types were assigned: malignant cells (43.4% total cells, purple), mesothelial (9.7%, orange), T cells (18.7%, dark blue), NK cells (9.7%, light blue), B cells (1.4%, yellow) and cell types belonging to a myeloid lineage (11.8%, green). Bar shows the proportion of each cell type across all seven MPEs. Cell‐type colours are used consistently throughout the figure. (B) Clustered correlations (spearman) between transcriptomic profiles of different cell types aggregated within each patient (see Supplementary Methods), showing evidence for whole‐transcriptome similarities across similar cell types. The colours of the cell types are to the same as Figure 1A. The same order was used in the X and Y axes. (C) Gene expression patterns across cell populations using the normalized expression of upregulated genes for each cell type, averaged within each patient (see Supplementary Methods). Hierarchical clustering of genes shows clear patterns of differential expression between cell types, annotated with marker genes of interest. (D) Cell‐type proportions per patient showing the diversity across patients with different disease subtypes. Each horizontal bar represents the cell type proportions in a patient and total cell number is indicated on the right‐hand side. Cell type according to colour legend in panel A. (E) Percent of cells in each cell type with non‐zero expression of nine marker genes across MPE and primary breast tumour^25^ datasets. Cell type according to colour legend in panel A, except for additional cell types in primary samples (CAFs and endothelial).

For the heatmaps in Figure 1, hierarchical clustering was performed on the genes using the hclust R library and scaled for heatmap visualization. The genes in the expression heatmap (Figure 1C) were curated by taking unique genes for each cell type based on features selected from the data using SingleR's Wilcox method, after excluding mitochondrial and ribosomal genes. Cell clusters with less than 100 cells in a cell‐type group were excluded. Overlapping genes for some cell types were included.

### 2.4 Processing of external datasets

Reference datasets for cell‐type annotation were downloaded from the NCBI Gene Expression Omnibus (GEO) database, including 20 304 cells from pleural fluid samples of lung adenocarcinoma patients^22^ (GSE131907), 9609 high‐grade serous ovarian cancer cells from malignant ascites samples^26^ (GSE146026) and 6734 cells in ‘Endothelial’, ‘Epithelial’ and ‘Stromal’ categories from 22 non‐smoker samples^27^ (GSE136831). For the lung and ovarian cancer datasets, we filtered lowly expressed genes from the provided normalized data. For data from Adams et al., we removed cell types with five or fewer cells leaving 18 cell types and log‐normalized as described above.

Seurat objects containing malignant cells from primary breast cancers for 13 ER^+^ and eight TNBC patient samples were downloaded from figshare.^28^ Objects contained gene counts and cell metadata used to generate figures for comparison between primary breast cancer and MPE samples.

## 3 RESULTS

### 3.1 Single‐cell RNA sequencing of breast cancer pleural effusions highlights diverse cellular compositions

To characterize the molecular and cellular make‐up of breast cancer MPEs, we collected the pleural fluid of seven late‐stage metastatic breast cancer patients who consented to donate their samples to the Olivia Newton‐John Cancer Research Institute‐Breast Cancer Biobank (ONJCRI‐BCB). Patients span various subtypes and treatment plans (Table S1), and all sequencing data were generated from post‐treatment pleural effusions, reflecting the patient‐level diversity that is typically observed in clinical settings. Two samples were from the luminal A subtype (BCB‐0114 and BCB‐0090), three luminal B (BCB‐0021, BCB‐0020 and BCB‐0139), including an inflammatory breast cancer sample (BCB‐0020), and two triple‐negative subtype (BCB‐0066 and BCB‐0112), including a BRCA1 mutated sample (BCB‐0066). This disease heterogeneity is echoed by the intra‐patient heterogeneity observed both in the cellularity of pleural effusions and the proportion of malignant cells present (Figure 1A,B). Substantial malignant cell populations were observed in four patients and each of these formed their own distinct clusters (Figure 1A), reflecting inter‐patient heterogeneity, whereas immune cell populations (T, NK, B and myeloid cells) produced clusters with a mix of patients (Figure S1) indicating similar phenotypes.

Across the experiment, cancer cells were the largest population overall (43.4% of total cells), followed by T cells (18.7%), myeloid cells (11.8%), NK cells (9.7%), mesothelial cells (9.7%) and B cells (1.4%) (Figure 1A). Cell‐type identities were determined using a consensus between automated annotations, classified using five independent datasets (Table S2), and manual annotations using marker genes for expected cell types of the pleural microenvironment. These cell‐type annotations were supported by transcriptome‐wide similarities between cell types without any batch correction, both in tSNE space (Figure 1A) and by clustering the average transcriptomic profiles per cell type from each patient (Figure 1B). Within cell types, we observed high correlations between the average transcriptomic profiles from each patient (Figure 1B), demonstrating phenotypic similarities between NK and T cells, across all immune cells, and between mesothelial and malignant populations.

Upregulated genes in each cell type show gene expression patterns consistent with cell‐type annotations and capture cell‐type differences and similarities (Figure 1C). For example, the upregulation of keratins (KRT7, KRT8, KRT18 and KRT19) and LMNA genes highlighted the expected similarities between mesothelial cells and breast carcinoma cells,^29^ while other genes central to mesothelial (WT1, MSLN, DES and CDH2) or epithelial (CDH1, CLDN4 and CLDN7) cell identity confirm their differences. Some B cell markers such as CD19 and CD27 were largely undetected in B cell populations, while other known B cell markers were upregulated (CD79A and IGHM), emphasizing the importance of using bioinformatics methods that are not reliant on marker‐based cell‐type assignment alone. Genes such as LTB and CD3D/G distinguished lymphoid cells from myeloid cell types, with few genes that overlap between these cell types across all patients (PTPRC/CD45 and some human leukocyte antigen [HLA] family genes). Natural killer (NK) cells and T cells, expected to be phenotypically similar, shared the expression of many genes (IL7R, IL32, CCL5 and CD2).

We found that in the absence of tumour‐cell enrichment or FACS, sequencing of pleural effusions capture varying malignant cell numbers (Figure 1D). In particular, some patients contained very few (< 50) malignant cells even though these patients (specifically BCB‐0090 and BCB‐0114) were treated after cytology reporting (Table S3). The remaining cell types (mesothelial, myeloid and lymphocyte populations) were found across all patients in varying numbers. In comparison, we processed and visualized scRNA‐seq data from 13 ER^+^ and eight triple‐negative primary breast tumours from the Pal et al. dataset,^23^ and found that primary breast tumours vary similarly in cell‐type proportion (Figure S2). Lymphoid and myeloid populations contributed to a substantial portion of each sample in MPE and primary breast tissue microenvironments, as opposed to CAFs, endothelial or mesothelial cells, which formed a smaller portion in one or the other location. We found mesothelial cells in similar proportions to the CAFs and endothelial cells in the primary samples, suggesting that these cells may play a similar role in the different tumour microenvironments. One notable difference between datasets is that, as expected, all primary breast cancer samples captured substantial malignant cell populations.

The gene expression patterns of cell type‐specific markers for malignant cells, T cells and B cells were consistent between the MPE and primary breast cancer samples (Figure 1E). Across the 112 684 cells analysed from primary breast tumours, a similar number of cancer cells expressed epithelial markers (EPCAM, CDH1 and ESR1). Other cell type‐specific markers were similarly detected in the primary tumours (CDH2, CD3D and CD79A), including a notably low detection of CD19 in B cell populations. However, mesothelial and NK cell markers found in the MPE were largely absent across the primary tumour dataset (DES and KLRF1; Figure 1E), reflecting the unique aspects of the pleural microenvironment.

### 3.2 Pleural effusion samples from different patients have a similar immune composition

**FIGURE 2:**
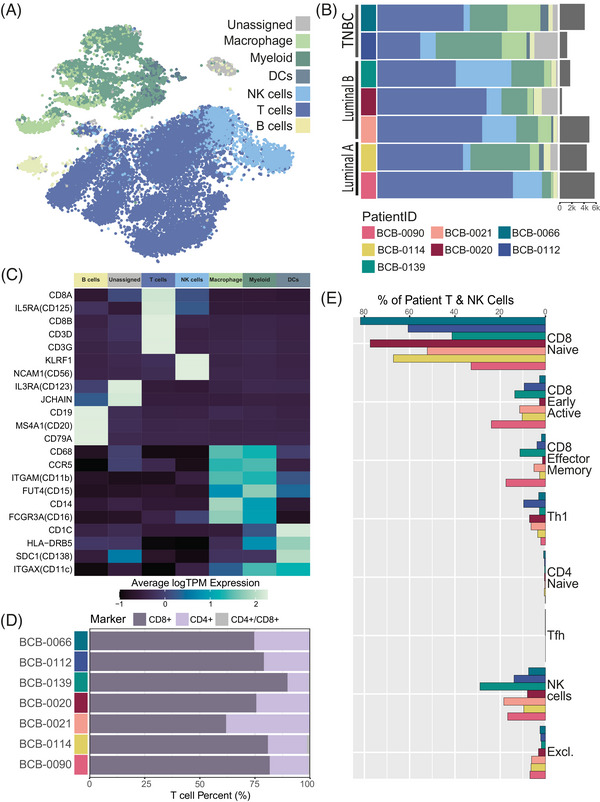
Immune components of malignant pleural effusions. (A) A tSNE of 22 414 immune cells from seven breast cancer patients coloured by cell type. (B) Diversity of patient immune profiles represented as the proportion of overall cells per patient for different disease subtypes. Each horizontal bar represents the cell‐type proportions in a patient and total cell number is indicated on the right‐hand side. Cell type according to colour legend in panel A. (C) Average normalized expression of marker genes across cell‐type populations. Hierarchical clustering of genes shows concordance between cell type‐specific marker genes and cell‐type identities. (D) Percentage of T cells that are CD4‐positive (light purple) or CD8‐positive (dark purple) in each patient. Across all patients, there are 31 double‐positive cells (both CD4‐ and CD8‐positive), which are included as grey. (E) Percentage of T and NK cells in each patient that belong to each subpopulation. Patient ID according to colour legend in panel B.

The pleural space is a source of immune‐competent cells during respiratory infections or during airway diseases.^30^ Mesothelial cells initiate immune responses in the pleura by recruiting inflammatory cells through the secretion of cytokines, chemotaxis regulation^31^, ^32^ and increased pleural permeability,^33^ allowing the transmigration of these cells into the pleural space.^30^, ^33^, ^34^ In addition, mesothelial cells may activate resident macrophages^34^ and form a mesothelial‐macrophage crosstalk that enhances a mesothelial‐driven immune response.^33^, ^34^ Given the inflammatory environment of pleural metastases, we expected a large presence of recruited immune cells in our non‐enriched MPE samples. In this metastatic setting, we identified distinct myeloid and lymphocyte populations (Figure 2A). Each cell type was detected in all patients and while cell‐type proportions were heterogeneous across patients (Figure 2B), there was no detectable pattern of subtype specificity (Figure S3).

While we could not reliably distinguish between monocytes and macrophages, we identified dendritic cell (DC) populations. Annotation of DC populations used a combination of reference datasets and negative selection based on genes that were expected to be absent in DCs (CD14, CD56 and CD15) (Table S2). This was supported by sub‐clustering of myeloid populations, where some DCs formed distinct clusters (Figure S4). As DCs are known to play a role in local inflammatory responses and innate immunity of the pleural cavity,^35^ the presence of DCs and effusion‐infiltrating lymphocytes suggests that the pleural fluid is an inflammatory micro‐environment for malignant cells.

Lymphocyte populations formed the largest portion of immune cells (71%) and expressed canonical marker genes, including T cell (CD8A, CD3D/G, CD25), NK cell (KLRF1, CD56) and B cell (CD19, CD20, CD79A) genes that were absent in myeloid populations (Figure 2C). NK cell proportions were considerably more variable than other cell types (Figure 2B), reflecting different immune responses across patients. While the molecular similarity between these cell types made them vulnerable to misclassification, NK annotations were consistent with the expression of discriminatory genes (KLRF1 and CD56). Additionally, a population of unassigned cells formed a strong cluster that could not be confidently labelled due to a general disconcordance of cell‐type labels and sparsity of canonical marker gene expression. Subclustering of these unassigned cells with B cell populations suggested a B cell‐like or plasma cell identity. This cluster showed low expression of some B cell markers (CD52 and IGHM) but upregulation of canonical marker genes (JCHAIN and IRF4) previously used to identify plasma cells (Figure 2C and Figure S5).^23^

Across non‐enriched samples, T cells were the most prominent immune cell type, making up 56% of immune cells. Frequencies of T cells and their subtypes play a significant role in determining the response to therapy and survival of patients.^36^ The relationship between patient‐specific differences and T cell heterogeneity in the pleural space is not well understood. Several studies have compared CD8^+^ and CD4^+^ T cell frequencies across matched MPE and tumour tissue, or MPE and peripheral blood samples, with conflicting results.^36^ Previous flow cytometry studies on mesothelioma and lung cancer patients suggest that CD4^+^ regulatory T cells are recruited to the pleura and report high proportions of CD4^+^ T cells relative to CD8^+^ cells in MPEs,^36^, ^37^, ^38^ where CD8^+^ T cell dominance has been observed in non‐MPEs.^39^ Of the 35% of T cells that expressed either CD8 or CD4 in our dataset, the majority were CD8^+^ T cells (Figure 2D). This CD8^+^ T cell dominance was stable across all patients, despite malignant population size, suggesting that it did not relate to the breast cancer subtype. The CD4^+^/CD8^+^ T cell ratio is substantially more variable across patients from the Pal et al. dataset, containing 19 treatment‐naïve primary breast tumours (Figure S6). This suggested either that the tumour microenvironment is less heterogeneous in the pleura than in the breast, or that the treatment had an impact on the heterogeneity of the immune repertoire.

Since only 35% of T cells expressed either CD8A/B or CD4, we assessed T cell heterogeneity in more detail. To do so, we performed subtyping of T cells using ProjecTILs, by projecting T cell populations into a reference dataset of annotated T cell subtypes.^24^ Consistent with the CD4^+^/CD8^+^ ratio, we found that the majority of T cells were CD8 positive (Figure 2E), where few cells had conflicting marker expression and classification (‘Excluded cells’ Figure 2E, see Supplementary Methods). While the subtypes seen within the CD8^+^ T cell populations were also relatively stable across patients and cancer subtypes, the observed variability between some patients – for example BCB‐0139 and BCB‐0066 – may be due to differences in treatment response. However, the majority of CD8^+^ T cells across patients were classified as naïve‐like CD8^+^ T cells and this likely reflects the post‐treatment status of the patients.

### 3.3 Inter‐patient heterogeneity of malignant and mesothelial cell populations reflects disease subtypes

**FIGURE 3:**
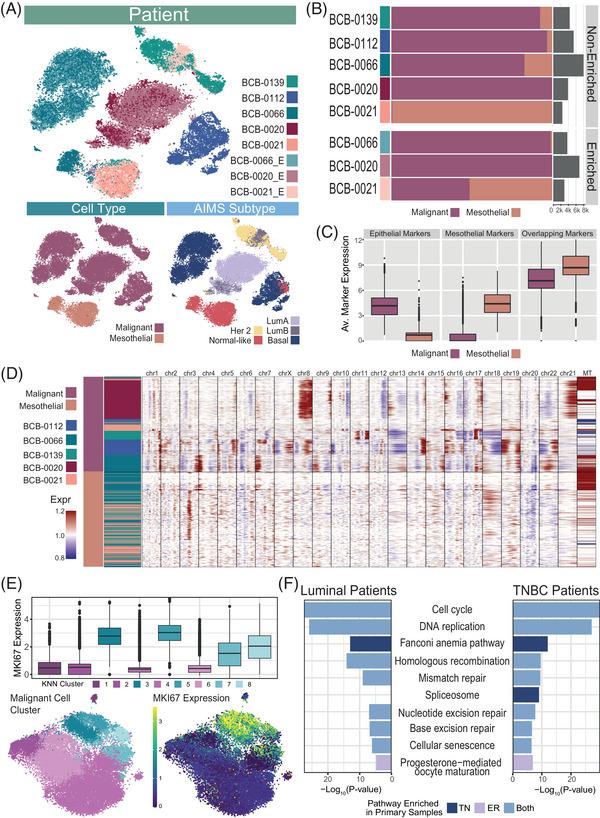
Inter‐patient heterogeneity in the mesothelial and malignant composition of MPEs‐level heterogeneity of cancer cell phenotypes. (A) A tSNE of 37 428 non‐immune cells from eight breast cancer patient samples (five non‐enriched, three enriched) coloured by (i) patient ID (E: enriched), (ii) cell type and (iii) breast cancer subtypes based on the AIMS breast cancer classifier on pseudo‐bulk samples. Cell type and subtype colour legends here are used throughout the figure. (B) Proportions and total number of malignant and mesothelial cells per patient, illustrating cancer cell presence is variable among patients, and that standard cancer cell enrichment strategies still retain mesothelial cells in MPE samples. Each horizontal bar represents the cell‐type proportions in a patient and total cell number is indicated on the right‐hand side. Cell type according to colour legend in panel A. (C) The average log2(TPM+1) expression of three groups of marker genes across malignant and mesothelial populations. Expression has been averaged across genes in each group, per cell. This includes epithelial breast cancer‐specific marker genes (CLDN4, IMP3, MUC1, CDH1, CEACAM5, SCGB2A2, CLDN3 and CLDN7) and mesothelial‐specific marker genes (CALB2, WT1, UPK3B, CDH2, COL1A2, S100A4 and MSLN) known to distinguish between malignant epithelial and mesothelial cell populations, in contrast to common overlapping markers (KRT8, KRT19, VIM, CD44 and ACTB) that make it difficult to distinguish these populations. (D) Malignant and mesothelial cell populations were subsampled from patient clusters (see Supplementary Methods), and the heatmap shows their expression intensity (based on read depth) across chromosomal positions relative to a baseline of benign epithelial mammary cells and non‐malignant immune cells from five patients. This can be used to identify regions of the tumour genome that may have undergone somatic copy number variation. (E) Analysis of 31 628 malignant cells from seven samples (BCB‐0020, BCB‐0139, BCB‐0112 and BCB‐066, and three enriched samples: BCB‐0020_E, BCB‐0021 and BCB‐0066_E). Patient data were integrated and clustered into eight clusters, as shown on the left tSNE. Clusters 3, 5, 7 and 8 (blue) show high MKI67 log‐expression, where the remaining clusters (1, 2, 4 and 6) have low or no expression of MKI67 (boxplot, right tSNE). (F) We pseudo‐bulked cells from the eight clusters in Figure 3E for luminal (BCB‐0020, BCB‐0139, BCB‐0020_E, BCB‐0021_E) and triple negative breast cancer (TNBC) (BCB‐0112, BCB‐0066, BCB‐0066_E) separately, then performed a differential expression between clusters with high MKI67 expression (clusters 3, 5, 7 and 8) and low MKI67 expression (clusters 1, 2, 4 and 6). KEGG pathways upregulated in the MKI67‐high clusters (blue), in either luminal (ER: Estrogen Receptor) or triple‐negative breast cancer patients (TN: Triple Negative), are presented with their corresponding −log_10_(*p*‐value). The horizontal bars indicating *p*‐value are coloured based on whether or not the pathway is also enriched in luminal or TN (or both) primary breast cancer samples.^23^

We next focussed on the heterogeneity of malignant and mesothelial cells. Overall, our dataset contained 33 972 malignant and 6626 mesothelial cells from eight samples (drawn from five patients). This included an additional 15 323 cells from the enriched samples of three patients (BCB‐0066, BCB‐0020 and BCB‐0021). We performed Miltenyi enrichment on these three samples using a negative selection approach. One sample had a low number of malignant cells (BCB‐0021) and two had a substantial proportion of malignant cells (BCB‐0066 and BCB‐0020). Unlike the immune cells that clustered by cell type (Figures 1B and 2A), malignant cells clustered by patient (Figure 3A), suggesting that there was substantial inter‐patient heterogeneity in these cell populations. Some heterogeneity was also observed in the molecular phenotypes of malignant cells from a single patient (intra‐patient heterogeneity).

We found that Miltenyi enrichment substantially increased the proportion of malignant cells in our dataset (Figure 3B). However, we observed the persistence of non‐malignant cells initially identified as fibroblast‐like cell populations (subsequently identified as mesothelial cells). after cancer cell enrichment. This prompted us to more precisely define the malignant population and seek a more definitive identification of the remaining non‐malignant cells.

Due to the phenotypic similarities between carcinoma cells and mesothelial cells, distinguishing between these cell populations is limited to a handful of non‐overlapping, discriminatory genes (Figure 3C, ‘Epithelial’ and ‘Mesothelial’ markers). Desmin and EpCAM are two commonly used marker genes that are capable of this distinction.^40^ In our dataset, 78.6% of malignant cells were EpCAM positive and 48.4% of mesothelial cells were Desmin positive (Figure S7). We found that breast cancer cells had a higher expression of epithelial markers (CLDN4, IMP3, MUC1, CDH1, CEACAM5, SCGB2A2, CLDN3 and CLDN7) that are associated with breast cancer,^21^ while mesothelial cell populations had a higher expression of known mesothelial markers (CALB2, WT1, UPK3B, CDH2, COL1A2, S100A4 and MSLN)^21^, ^41^, ^42^ (Figure 3C, ‘Epithelial’ and ‘Mesothelial’ markers). Specifically, the mesothelial genes used here have been established as markers capable of distinguishing between benign mesothelial populations and carcinoma cells.^4^ As expected, both cell populations expressed similar levels of overlapping markers (KRT8, KRT19, VIM, CD44 and ACTB)^14^, ^43^, ^44^ (Figure 3C, ‘Overlapping’ markers) that are key to both epithelial and mesothelial cell‐type identities. Notably, shared markers VIM and CD44 are also markers of mesenchymal‐like cells.

To confirm this, we next inferred copy number variations (CNVs) from gene expression data to distinguish malignant cells, based on their genomic instability, from normal cells (in this case, mesothelial cells).^45^ Relative to a baseline of benign mammary epithelial cells^46^ and known non‐malignant cells from these patients (immune cells), we observed large‐scale transcriptomic differences between malignant and mesothelial cell populations that may indicate somatic CNVs (Figure 3D). These proposed CNVs were consistent with previously identified CNVs in bulk DNA‐sequencing breast cancers,^47^ such as copy number gains on chromosome 8q and chromosome 17q, which are consistent across most of the patients (Figure 3D). This evidence of extensive rearrangement was absent from the mesothelial cells in the lower part of Figure 3D, which appeared to be more ‘normal’ in their putative genomic structure.

To explore the phenotypic heterogeneity of cancer cell populations, both between and within patients, we applied pseudo‐bulk analyses to enable the classification of samples into breast cancer subtypes. For each patient, we clustered non‐immune cells, and then aggregated clusters of cells into pseudo‐bulk samples to which we applied the Absolute Intrinsic Molecular Subtyping (AIMS) classifier.^48^ Although AIMS was initially designed for bulk‐sequencing data, we observed that subtypes assigned to malignant clusters were largely concordant with the annotated breast cancer subtype of the patient's primary tumour (Figure 3A, Figure S8, and Table S1). For luminal B patients with sufficient malignant cells (BCB‐0139, BCB‐0020 and BCB‐0021; Figure 3B), we observed a mix of luminal A, luminal B and Her2 subtypes across pseudo‐bulk clusters. To further assess the diversity of molecular subtypes from the two TNBC patients, we applied gene sets defined by Lehmann et al.^49^ to malignant cells from BCB‐0112, BCB‐0066 and BCB‐0066_E (see Supplementary Methods). Although AIMS classifies clusters from these samples as basal, we observed that these patients were phenotypically heterogeneous according to the TNBC Lehmann subtypes (Figure S9). These results showed that scRNA sequencing of MPEs, while largely concordant with primary tumour subtype classifications, can identify molecular heterogeneity with high resolution.

Mesothelial cell populations were included in our AIMS subtyping to investigate their role in the phenotypic diversity of liquid biopsies from MPEs. All mesothelial clusters and a small number of malignant clusters were classified as normal‐like in our pseudo‐bulk analysis. The PAM50 normal‐like classification was developed using normal breast tissue^50^ and it is expected that cancer samples contaminated with normal tissue will classify as normal‐like.^51^ This ‘normal’ class was refined for the AIMS subtyping model, where more samples switched to a normal‐like classification due to an increase in the signal of non‐neoplastic gene expression.^48^ While this classification may not always represent normal tissue, we expected that cells with fibroblast‐like phenotypes would classify as normal‐like. In addition, the ‘normal‐like’ gene expression pattern was characterized by a low expression of typical luminal epithelial genes,^51^ characteristic of these pleural mesothelial populations.

We also performed pseudo‐bulk analyses on malignant cell clusters to compare malignant subpopulations found in MPE to those previously identified in primary breast cancer patients. We integrated malignant cells from each patient to obtain clusters that were common across patients (Figure S10). We identified a distinct MKI67‐positive proliferative subpopulation (Figure 3E) that resembled similar MKI67‐positive clusters identified by Pal et al. in luminal and TN primary breast cancer samples.^23^ To demonstrate similarities between these MKI67‐positive subpopulations, we performed gene set enrichment analyses on pseudo‐bulked samples that were obtained by aggregated malignant cells from each cluster (in Figure 3E) for each patient. Differential expression analyses were performed separately for pseudo‐bulk samples from the luminal (BCB‐0020, BCB‐0020_E, BCB‐0021 and BCB‐0139) and TNBC patients (BCB‐0112, BCB‐0066 and BCB‐0066_E) and showed that the enriched phenotypes were captured by both subtypes (Figure 3F). Pal et al.^23^ indicated 13 KEGG pathways that were enriched in the proliferative cluster of ER^+^ or triple‐negative breast cancer patients. Most of these were found among the top 20 enriched KEGG pathways in our differential expression analyses of luminal or TNBC patients (Figure 3F), including cell cycle, DNA repair pathways and pathways that contain proliferation‐associated genes. Notably, one of the three KEGG pathways that were enriched in primary breast cancer samples but not our MPE samples was oxidative phosphorylation, which may relate to the impaired oxygen supply expected in the pleura. Similarly to Pal et al., the MKI67‐positive subpopulation was more dominant in TNBC samples in our dataset (Figure S10) and may represent a reservoir of proliferating malignant cells that can persist through tumour progression and in different microenvironments.

**FIGURE 4:**
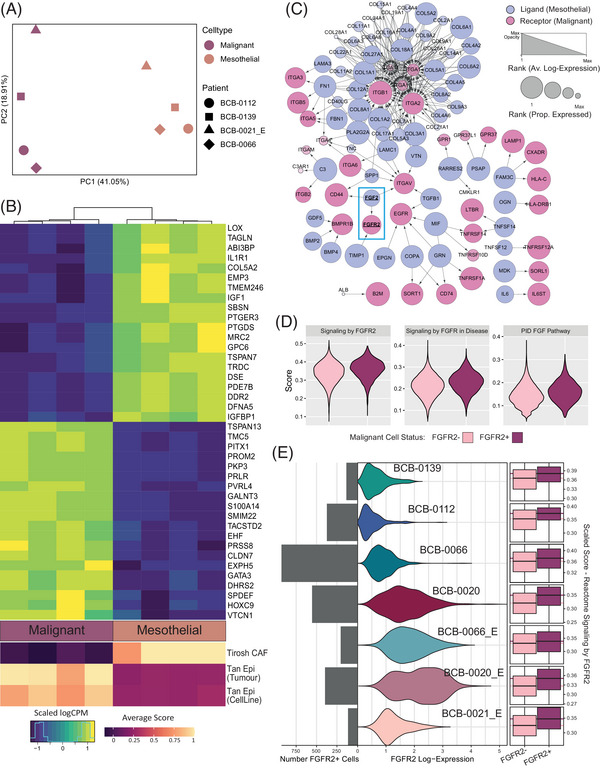
Mesothelial‐derived CAFs in malignant pleural effusion. (A) PCA plot demonstrating transcriptomic differences between pseudo‐bulked malignant (pink) and mesothelial (orange) populations. Pseudo‐bulk samples are aggregated within each patient for patients with sufficient numbers of malignant and mesothelial cells (BCB‐0112; circle, BCB‐0139; square, BCB‐0066; diamond, BCB‐0021 [enriched]; triangle). (B) Upper heatmap shows the scaled log(CPM) (counts per million) expression of 40 differentially expressed genes (corrected *p*‐value < .05, FDR < .05), including the top 20 genes upregulated in malignant pseudo‐bulk samples (pink) and top 20 genes upregulated in mesothelial pseudo‐bulk samples (orange). The lower heatmap shows the average gene set score across malignant (pink) and mesothelial (orange) cells in each pseudo‐bulk sample for a CAF signature and two epithelial signatures to describe the CAF phenotype of mesothelial cells and the relative epithelial phenotype of malignant cells. (C) Network representation that captures the top 100 interactions between ligands and receptors that are expressed in mesothelial (pink) and malignant (purple) cell populations, respectively. Node size indicates the proportion of cells in each cell type that express a gene, and transparency shows average log‐expression. (D) Gene set scores for FGFR2‐positive (light pink) and ‐negative (dark pink) malignant cells for FGF‐related signalling pathways. (E) Expression of FGFR2‐positive malignant cells across patients compared with per‐patient gene set scores. Total number of FGFR2‐positive cells per patient is indicated by the bar chart (left). The log‐expression of FGFR2 in FGFR2‐positive malignant cells across patients is shown in the middle. The boxplots (right) summarize gene set scores for FGFR2‐positive (light pink) and ‐negative (dark pink) malignant cells in each patient. The light pink and dark pink horizontal lines through the boxplots indicate the median score for FGFR2‐negative and ‐positive cells, respectively.

While the literature supports a CAF‐like role for pleural mesothelial cells, it is suggested that this occurs following MMT.^14^, ^16^ However, the transcriptomic signatures of mesothelial cell populations in our dataset suggested a CAF‐like mesothelial phenotype that may be supporting tumour growth, while retaining their mesothelial identity. First, we characterized mesothelial and malignant populations by performing a differential gene expression analysis using pseudo‐bulk approaches (Table S4). To reduce noise introduced by potentially misclassified cells, we excluded cells that lacked the expression of key cell‐type markers (see Supplementary Methods). These markers were selected based on their prevalence in our dataset (Figure S7) and performance in cytology‐based studies.^12^, ^40^, ^41^, ^42^ A principal components analysis (PCA) showed that cell type was the main source of variation between pseudo‐bulked samples (principal component 1; Figure 4A) and that mesothelial cells from different patients were more similar to one another than they are to the malignant populations. The top 20 up‐ and down‐regulated genes between malignant and mesothelial populations showed expected markers, such as PVRL4 or COL5A2, as well as genes that suggested a metabolically active cancer cell phenotype (WNT7B) and reactive mesothelial cells (MMP2 and LRP1) (Figure 4B, upper heatmap).

We then performed gene set enrichment analyses and found that mesothelial cells were enriched for gene sets relating to vascularization, pro‐angiogenic phenotypes, and ECM remodelling across GO, KEGG and Hallmarks gene sets (Table S5). Whereas cancer cell populations were enriched for a proliferative phenotype, including cell cycle (*KEGG, GO, Hallmarks*), metabolism (*GO*) and protein secretion (*Hallmarks*). Together with the differential expression of growth factors (e.g. fibroblast growth factor 2 [FGF2], vascular endothelial growth factor C [VEGFC], platelet derived growth factor D [PDGFD] and insulin‐like growth factor 1 [IGF1]) by the mesothelial cells, these results would suggest that pleural mesothelial cell populations could play a CAF‐like role supporting the survival of cancer cells without undergoing MMT.

Pseudo‐bulk approaches allow us to apply well‐established bulk RNA‐seq analysis methods; however, they are new and sensitive to cluster size. To complement this analysis, we also applied a single‐cell scoring method^52^ to assess potential CAF phenotypes of mesothelial cells. We scored individual cells with a previously derived CAF signature^53^ and two epithelial breast cancer gene sets^54^ (Figure 4B, lower heatmap). The averaged cell score per patient was compared with the differential expression results from the pseudo‐bulk analysis (Figure 4B, lower heatmap), showing that malignant populations display cancer‐associated gene expression and mesothelial populations consistently express a CAF‐like phenotype. These results provide further evidence of a CAF‐like role that is supporting the progression of the cancer.

Next, we constructed a ligand‐receptor network to explore potential interactions between mesothelial cells and malignant cells in MPE samples, and to investigate the mechanisms underpinning their CAF‐like relationship. The top 100 ligand‐receptor pairs from this analysis (Figure 4C) were differentially expressed in the pseudo‐bulk analysis, including collagens, integrins and FGF2, and belong to some of the ECM remodelling and vascularization gene sets identified as enriched. While studies report mesothelial cell‐derived CAFs that promote the survival of malignant cells,^13^, ^14^ our results suggest that mesothelial cells might interact with malignant populations to support their growth through a CAF‐like phenotype.

We focused on ligand‐receptor pairs where the ligand was expressed by the mesothelial cells but not by the cancer cells, pointing to a directed flow of information from mesothelial cells to cancer cells (Figure 4C) to identify cancer phenotypes most likely to be mesothelial‐induced. Leveraging the single‐cell resolution of our data, we split cancer cells into FGF2 receptor (FGFR2) positive and negative cells and confirmed that differences observed in FGF2 expression were not due to the library size of the cells (Figure S10E). We then used pathway analysis to determine whether FGFR2‐positive cells have evidence for active FGF2 signalling (Figure 4D). Three downstream signatures of active FGF2 signalling were all more highly expressed in FGFR‐positive cells, despite these cells not expressing FGF2 itself. While the expression of FGFR2 varies across patients (Figure 4E), FGFR2‐signalling pathway scores are consistently elevated in FGFR2‐positive cells. As several studies have reported that FGF2 signalling promotes breast cancer growth,^55^, ^56^ our results pointed to FGF2 signalling as one potential mechanism for CAF‐like support of cancer growth by pleural mesothelial cell populations.

## 4 DISCUSSION

Cancer cell enrichment and flow cytometry are routinely used to select cell populations prior to single‐cell sequencing but may lose valuable insights offered by the phenotypes of the excluded populations and fail to capture the heterogeneity of the sample. Overall, we observed strong inter‐patient heterogeneity in terms of both cell‐type proportions and the cellularity of effusions that could be due to a large range of intrinsic and extrinsic parameters, such as the molecular characteristics of the tumours and the patient's medical history. Intra‐patient heterogeneity was also observed, and with a larger cohort of patients, studies of this type may help to interpret the pathophysiology of their metastatic disease and identify appropriate treatments.

Here, we showed that while lymphocyte and myeloid populations were found within all patient MPEs (Figure 1), some patient samples contained only a small numbers of malignant cells (BCB‐0021, BCB‐0114 and BCB‐0090), despite cytology‐positive effusions at earlier time points (Table S3). This illustrates the difficulties associated with isolating malignant cells from pleural fluid. We found that the cellular landscape of MPEs was similar to primary breast cancer with respect to the presence of infiltrating lymphocyte and myeloid populations (Figures S2 and S6), and even recapitulated a distinct MKI67‐positive proliferative subpopulation (Figure 3E and F).

The expression of key genes (CD3G, CD8A, CD79A, CD74, CD14 and CD16) (Figure 2C) supported the large presence of lymphocytic and myeloid populations, potential diversity in B cell populations (Figure S5) and revealed the dominance of CD8^+^ T cells across MPE samples (Figure 2D and E). While we observed a large presence of diverse lymphocyte populations, including a range of T cell subtypes, such as CD8 naive‐like, CD8 early active, CD8 effector memory, Th1, CD4 naive‐like and Tfh (Figure 2E), CD8 Tex, CD8 Tpex and Treg were not detected in MPEs. Although the proportions of immune cells relative to non‐immune cells described in our dataset are higher than in previously described lung pleural effusions,^10^ it will be interesting to confirm the proportion of the different cell subsets in MPEs using other methods, such as flow cytometry and immunohistochemistry, and analyse a larger number of cells. Furthermore, differences in the abundance of immune subtypes could be due to the tumour type and previous treatments, and the analysis of a larger number of samples will be essential to answer this question. Regardless, the high presence of lymphocyte populations across all patients indicates infiltration and increased capillary permeability. It is unclear whether this is in response to inflammation of the pleura, or the direct cause of entry for the malignant cells; however, these immune cell phenotypes reveal a highly inflammatory micro‐environment.

In addition to malignant and immune cell populations, we identified large proportions of mesothelial cells in some samples that appeared to be promoting tumour growth. Indeed, mesothelial cells are known to form spheroid clusters with ovarian malignant cells, providing support that enables the survival of cancer cells in the hypoxic environment such as ascites.^15^, ^57^ A key challenge arising from the clustering of malignant and mesothelial cells is the difficulty in distinguishing between these cell populations due to their similar phenotypes, morphologies, sizes and marker genes.^40^, ^41^, ^42^ For instance, VIM and CD44 are associated with mesenchymal phenotypes and are expressed by both mesothelial and carcinoma cells.^43^, ^57^, ^58^ Mesenchymal‐like cancer cells may have improved invasion of mesothelial monolayers^15^ and mesothelial cells can undergo a mesenchymal transformation when their monolayer is invaded.^20^ The phenotypic plasticity of mesothelial cells and metastatic carcinomas complicates cell‐type identification in the study and diagnosis of MPEs, making them prone to misclassification. Our study shows that mesothelial cell populations persist following standard cancer cell enrichment strategies, which would have implications in studies that do not appropriately annotate mesothelial cells following enrichment. Using consensus cell‐type annotation, the expression of discriminative marker genes, inferred CNVs and patient subtype‐specific gene expression patterns, we were able to differentiate between pleural mesothelial and breast carcinoma cells across all our breast cancer patients, and derived a distinctive gene expression pattern that captured a CAF‐like phenotype in pleural mesothelial cells (Figure 4A and Table S4).

After further refining cell‐type annotations, we investigated the potential role of mesothelial populations in MPEs. The pleural cavity is known to be conductive of mesothelium attachment and tumour establishment. It offers access to nutrients from adjacent stromal capillaries, offers an inflammatory environment that promotes pleural vascular permeability and angiogenesis, and provides mesothelial‐secreted growth stimuli. This inflammation and tumour vascularization, through factors like TNF^59^ and VEGF,^60^ plays an important role in MPE formation^61^ and could be therapeutically targeted to reduce pleural fluid volume. Mesothelial cells are primed to respond to inflammation, promote healing, and may even play an active role in the recruitment of inflammatory immune cells into the pleural space.^30^, ^33^ Cancer‐induced inflammation triggers mesothelial cells to convert into fibroblasts via MMT,^13^, ^16^ contributing CAFs to the tumour microenvironment, and mesothelial cell cultures have a demonstrated ability to form spheroid‐like clusters with cancer cell cultures, promoting proliferation and allowing survival in suspension.^57^ While a small number of mesothelial‐annotated cells may be fibroblasts, our analyses suggest that a CAF‐like phenotype is present in mesothelial cells without full conversion to a fibroblast phenotype. This supports the claim that at least some CAFs in the pleural metastatic niche may be of mesothelial derivation, but also suggests that the CAF‐like phenotype is present prior to fibroblast conversion.

The interactions between mesothelial cells and malignant cells that establish the metastatic niche are reported across multiple cancers.^13^, ^16^ However, the directionality of these interactions and their environmental context are poorly understood.^13^ Some studies have suggested that malignant cells in the pleura induce this phenotype in mesothelial cells to increase monolayer permeability, while others suggest that mesothelial cells may secrete factors that support tumour growth.^14^, ^19^ We identified FGF2 signalling as a possible mechanism through which mesothelial cells could be promoting tumour growth. While our analysis does not exclude the possibility that malignant cells can induce pleural mesothelium permeability, we found that mesothelial populations express genes encoding growth factors that may actively support cancer cell survival and proliferation in the pleural microenvironment.

FGF2 is an angiogenic factor that is associated with inflammatory responses, such as pleural fibrosis.^62^ While FGF2 signalling is essential for wound healing and normal development, the activation of FGF2 signalling in cancer cells can induce a proliferative, aggressive phenotype and is a target of various cancer therapeutics.^62^, ^63^ In breast cancer, FGF signalling may have differential effects based on hormone receptor status. For example, triple‐negative cell lines may be more sensitive to FGFR inhibitors,^55^ including two that harbour FGFR2 amplifications.^64^ Hormone‐responsive breast cancers are also receptive to FGF signalling, where studies have shown that FGF overexpression can promote oestrogen‐independent proliferation in ER^+^ breast cancers,^65^ and that signalling by FGFR2 may confer resistance to tamoxifen through degradation of ER,^56^, ^66^ or even that oestrogen may mediate FGF paracrine activation.^67^ While CAF‐secreted FGF2 has been shown to induce proliferation in breast cancer cell lines, it can be difficult to identify the origin of FGF ligands due to the observation of autocrine FGF signalling in breast cancer.^68^ Our data suggest that FGF2 signalling is a possible mechanism for mesothelial populations to provide a CAF‐like role to cancer cells, supporting the growth of cancer cells that have migrated to the pleura from the breast. Further investigations using long‐term co‐culture assays, gene editing and specific inhibitors and larger datasets will be required to further validate the involvement of this pathway, and to identify potential cellular or molecular markers associated with drug resistance.

## 5 CONCLUSIONS

Here, we present the first unbiased cellular landscape of MPEs from breast cancer patients. Our data provide a new depth in our understanding of the cellular phenotypes found in liquid biopsies of pleural metastases and how these cell populations may interact. In this unique tumour microenvironment, we observe intra‐patient heterogeneity of metastasized breast cancer cells from luminal and triple‐negative subtypes. We were able to identify a range of cytotoxic immune populations and mesothelial cell populations that display CAF‐like phenotypes. An improved understanding of the formation of MPE and the identification of the growth factors supporting the survival of breast cancer cells in the pleural cavity will inform the development of therapeutics to prevent or reduce metastatic disease.

## AUTHOR CONTRIBUTIONS

H.J.W. analysed the data and produced the figures and tables. H.J.W., J.B., M.J.D. and D.M. interpreted data and prepared the manuscript for submission. J.B., R.L.A. and D.M. designed and performed experiments. S.M. and A.T.P. provided input on the CNV analysis. C.B. and B.Y. provided samples and annotated clinical features. D.M. and M.J.D. supervised the project. All authors read and approved the final manuscript.

## FUNDING INFORMATION

D.M. and B.P. are supported by the NBCF (Investigator Initiated Research Grant IIRS‐19‐082). D.M., B.Y. and R.L.A. are supported by the Love Your Sister Foundation. D.M. is supported by Susan G. Komen and Cancer Australia (CCR19606878), the Victorian Cancer Agency Mid‐Career Research Fellowship (MCRF21011) and the Australian National Health and Medical Research Council (Grant 2012196). D.M., M.J.D. and B.Y. are supported by the Grant‐in‐Aid Scheme administered by Cancer Council Victoria. The contents of the published material are solely the responsibility of the individual authors and do not reflect the views of Cancer Australia or other funding agencies.

## CONFLICT OF INTEREST STATEMENT

The authors declare no conflict of interest.

## ETHICAL APPROVAL STATEMENT

This project was approved by Austin Health Human Research Ethics Committee, approval number HREC/14/Austin/425.

## Supporting information

Supporting Information

## Data Availability

The dataset supporting the conclusions of this article is publicly available in the Gene Expression Omnibus (GEO) repository under the accession GSE208532. The R scripts used to process and analyse these data, and to produce the figures presented in this paper, are available at https://github.com/hwhitfield/MPE_analysis_scripts.
