# Supplementary material for: Single‐cell RNA sequencing captures patient‐level heterogeneity and associated molecular phenotypes in breast cancer pleural effusions
Source: Clin Transl Med. 2023 Sep 10;13(9):e1356. doi: 10.1002/ctm2.1356 (PMC10493486; doi:10.1002/ctm2.1356)
Supplement: Supplementary file 1 — Supporting Information [file CTM2-13-e1356-s001.docx]

**
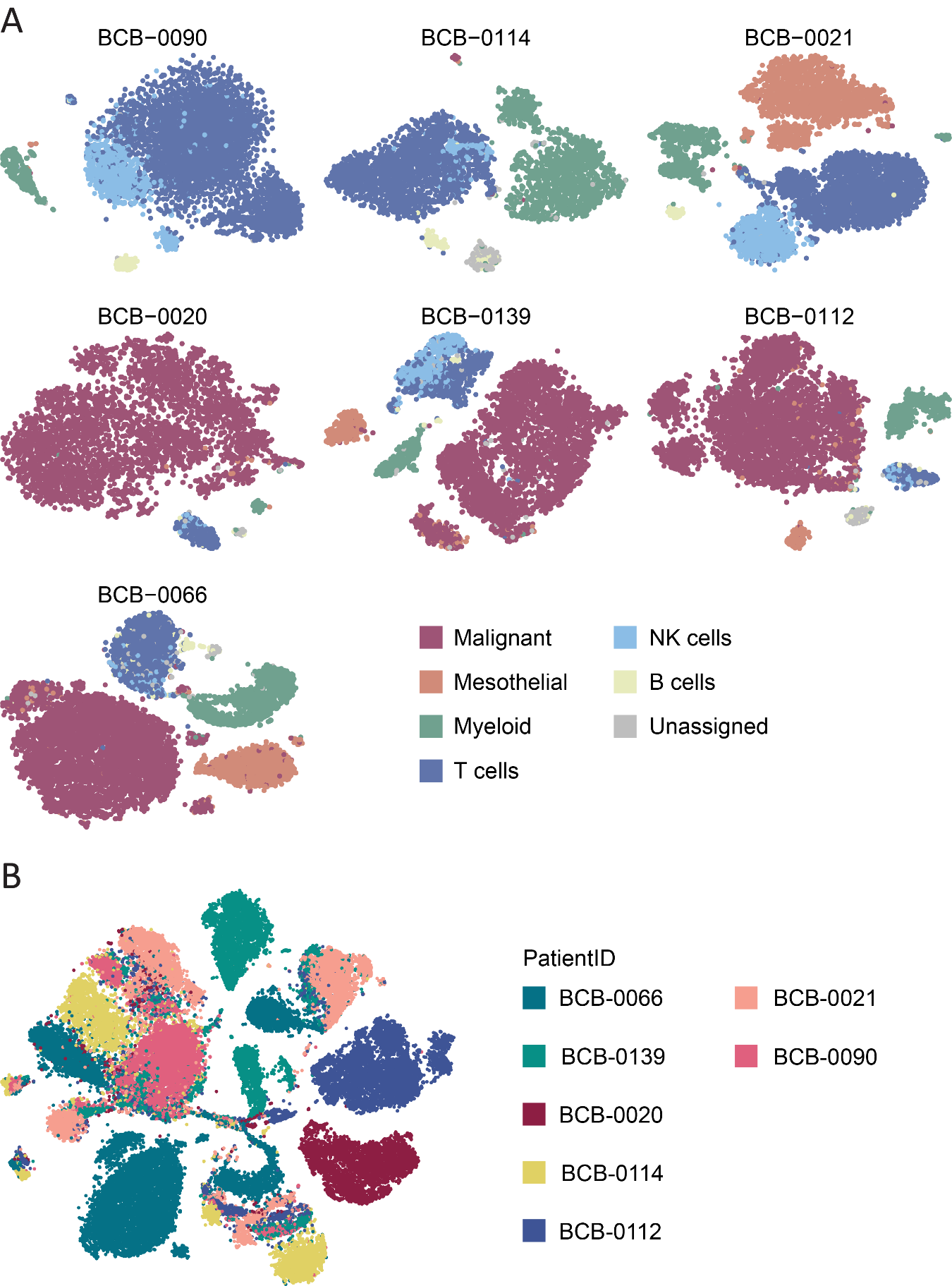
**

**Supplementary Figure 1 – All cell tSNE, per patient**

### A: Series of tSNE showing the distribution of different cell types in each patient. B: A tSNE of all cells from patients as seen in Figure 1 but coloured by patient ID.

###
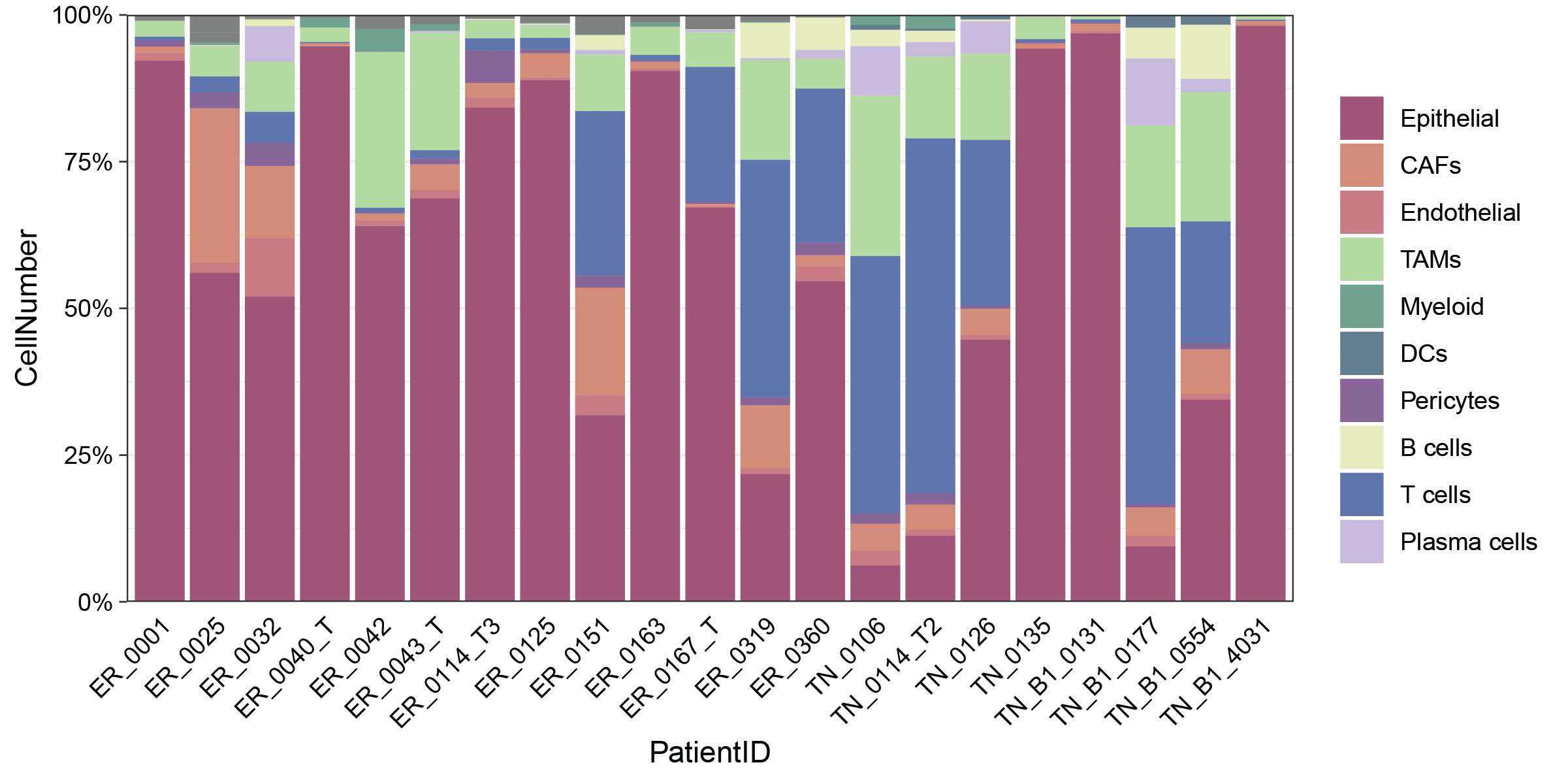


### Supplementary Figure 2 – Cell type proportions, primary breast cancer samples

Cell type proportions per patient from primary breast cancer samples 28, where each bar represents the cell type proportions in a patient. “PatientIDs” indicate ER+ (Estrogen Positive) and TN (Triple Negative) patients. Note that cell type colour legend is slightly different to the colour scheme used in the main figure.


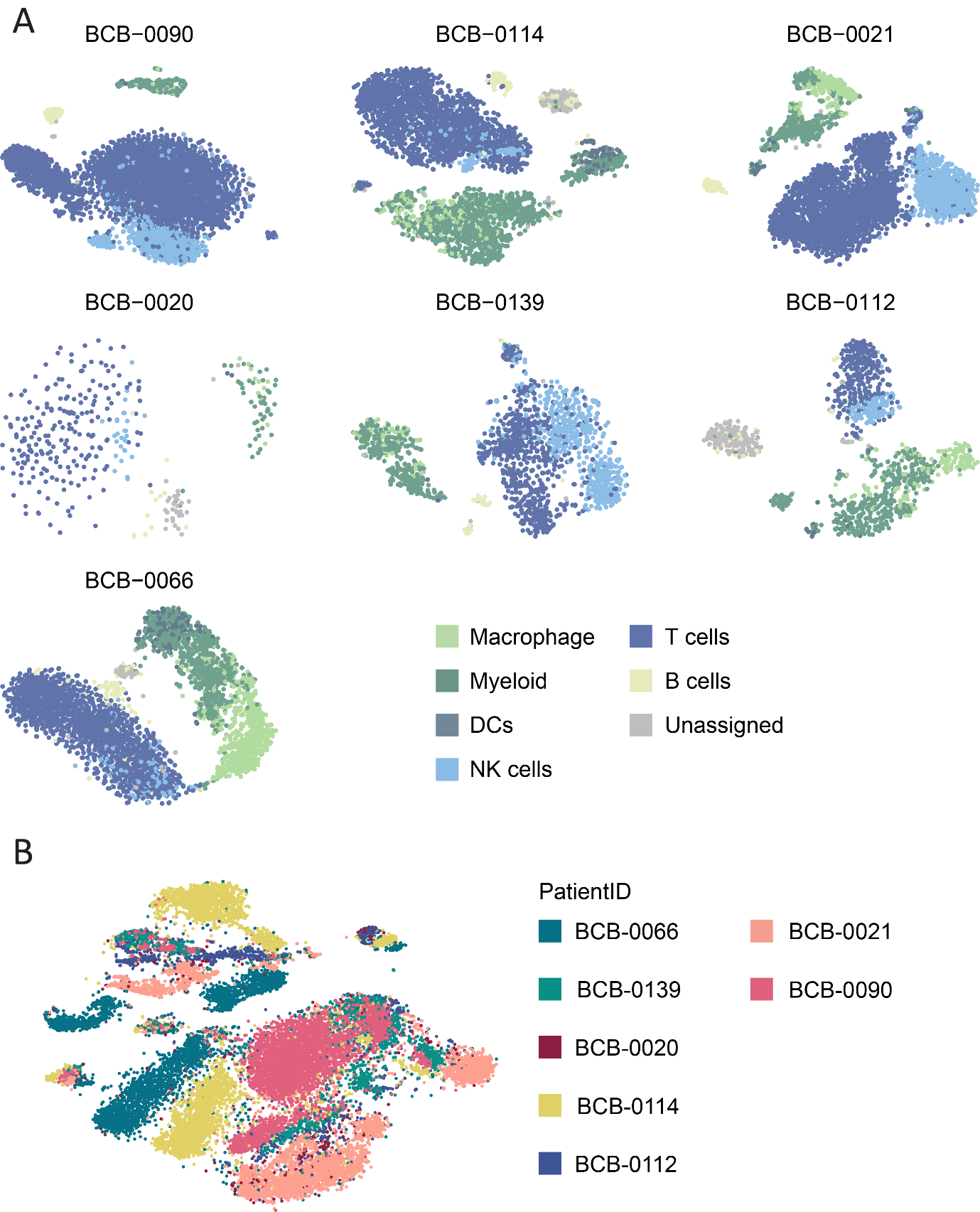


### Supplementary Figure 3 – Immune cell tSNE, per patient

A: The cell populations from Figure 2 visualised as tSNE for each patient, coloured by immune cell type. B: A tSNE of all immune cells from patients as seen in Figure 2 but coloured by patient ID.


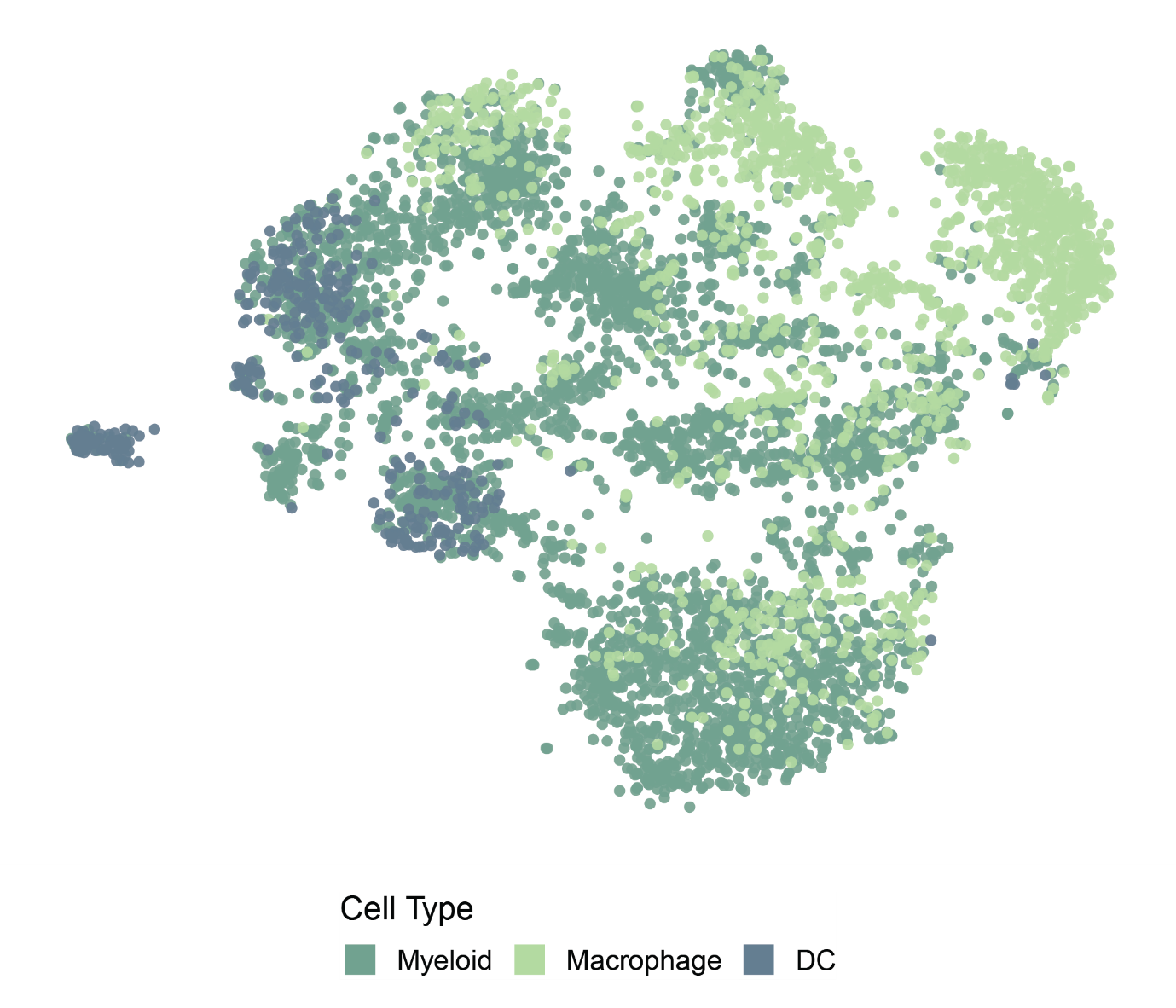


### Supplementary Figure 4 – Myeloid lineage tSNE

A tSNE for all myeloid-lineage cells per patients coloured by cell type, showing that DCs are transcriptionally distinct from other myeloid populations.


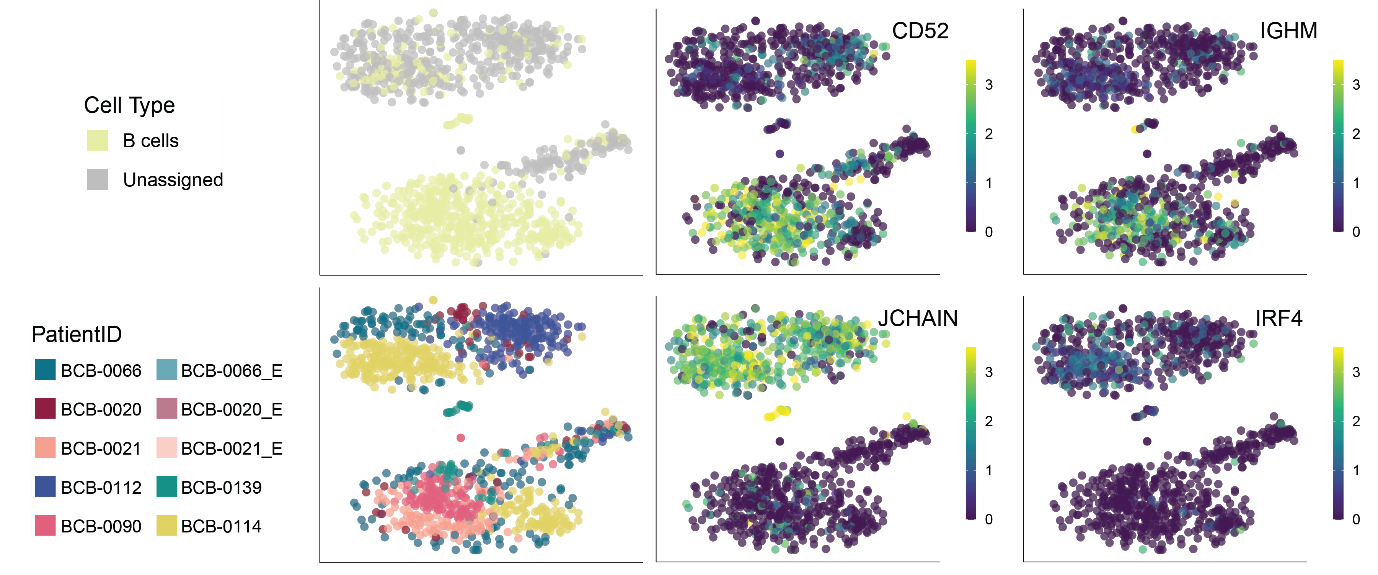


### Supplementary Figure 5 – Unassigned and B cell tSNE

A tSNE of B cell populations (yellow; 585 cells) and unassigned cluster (grey; 518 cells) coloured by cell type (i), patient (ii), CD52 (iii), J Chain (iv), IgM (v), and Interferon regulatory factor 4 (vi).


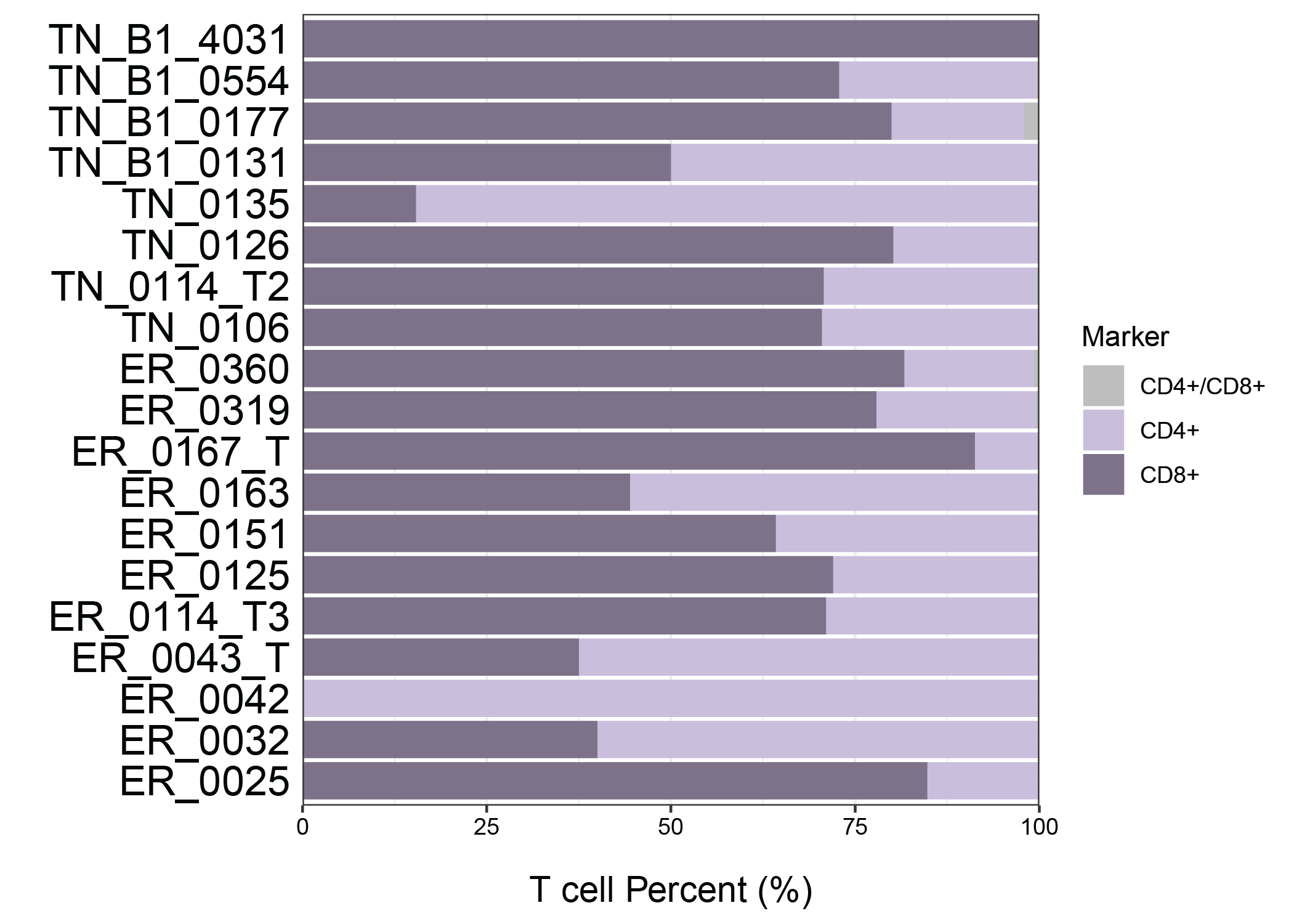


### Supplementary Figure 6 – CD4+/CD8+ ratio, primary breast cancer samples

Percentage of T cells from primary breast cancer patients ^28^ that are CD4-positive (light purple) or CD8-positive (dark purple) in each patient. Double-positive cells (both CD4- and CD8-positive) are included as grey.


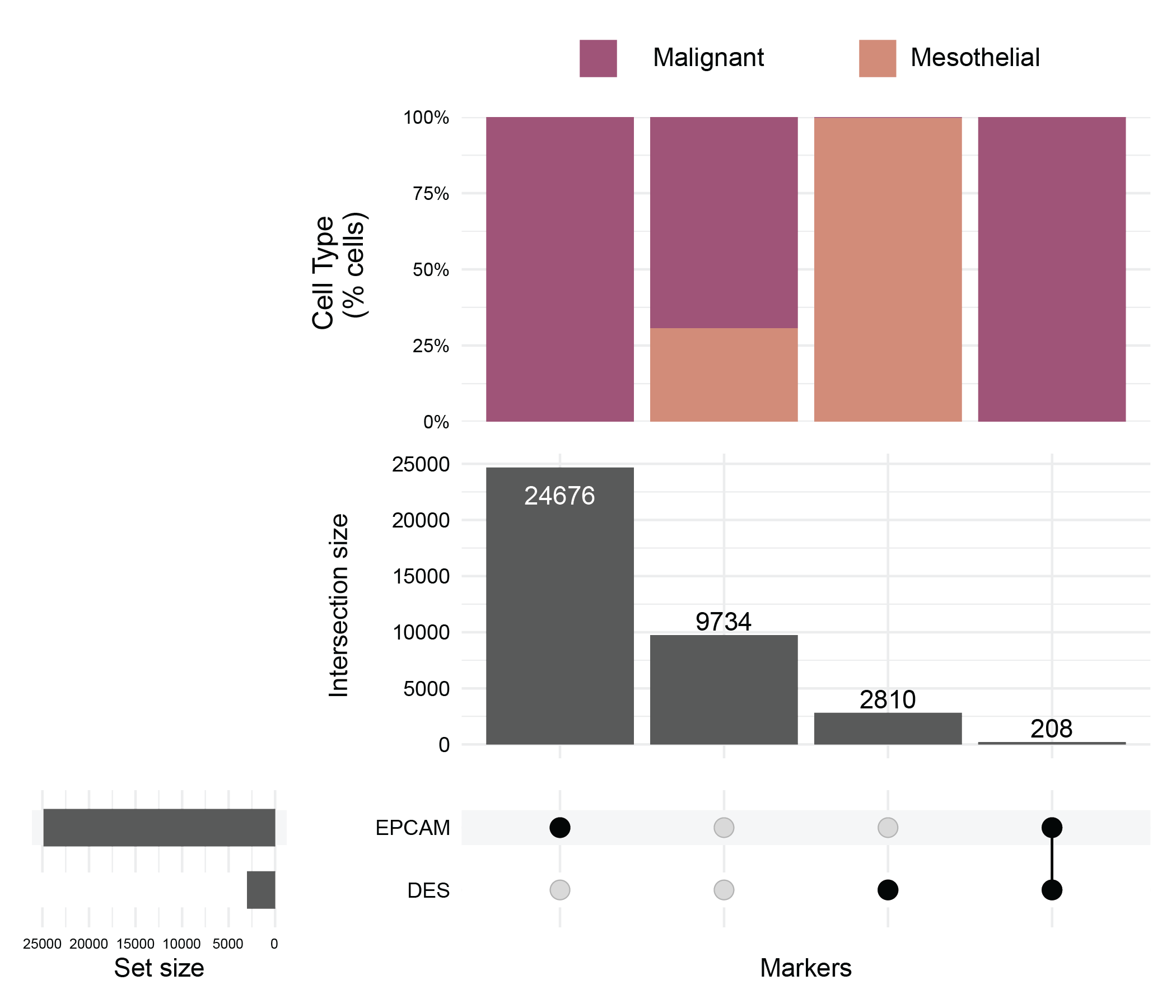


### Supplementary Figure 7 – Upset plot

An upset plot showing the expression of Desmin and EpCAM in mesothelial and malignant cell populations. Out of 31,640 malignant cells, 24,884 (78.6%) of those are EpCAM positive and 7.2% of all Desmin-positive cells are malignant.


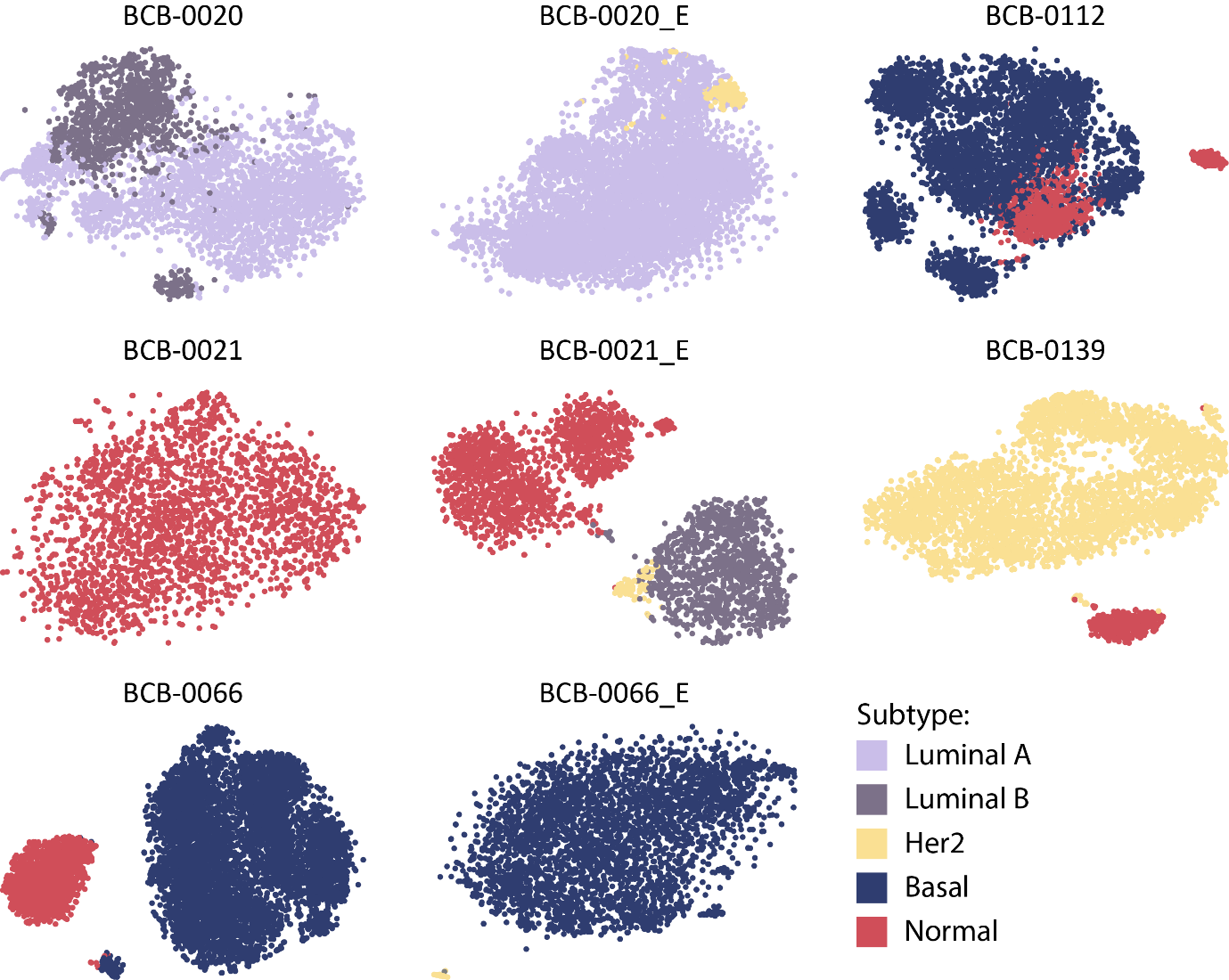


**Supplementary Figure 8 – AIMS Subtypes, tSNE per patient**

A tSNE per patient, coloured by breast cancer subtypes based on the AIMS classifier on pseudo-bulk samples.


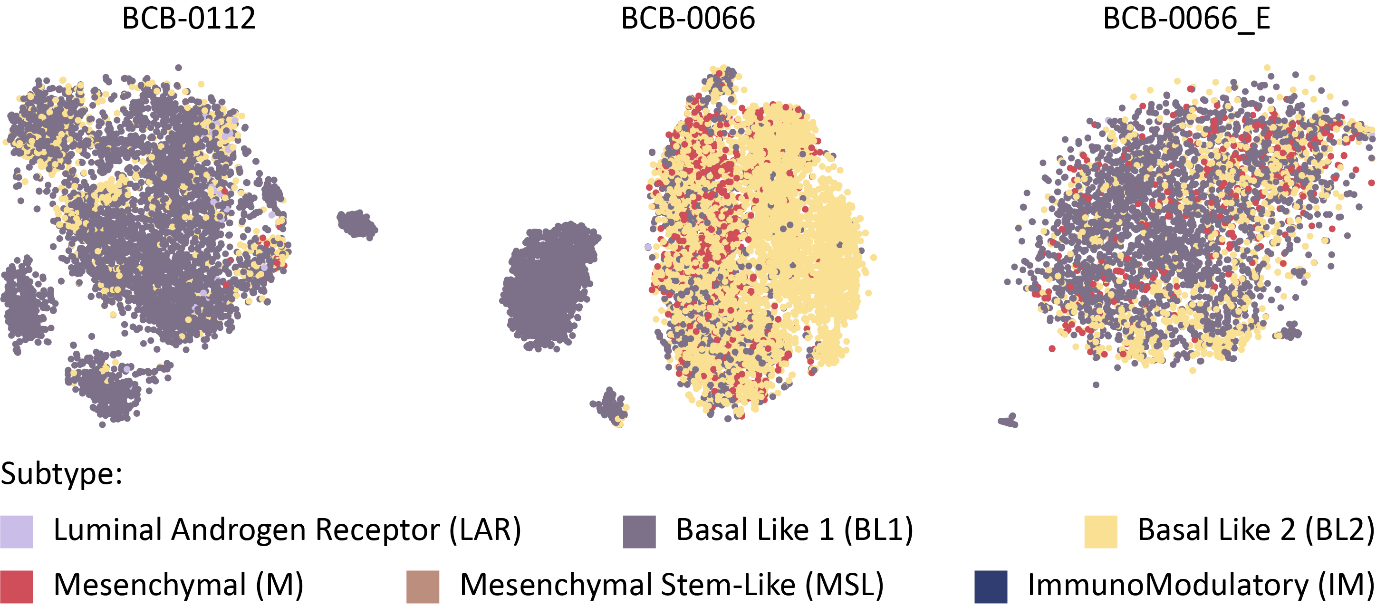


### Supplementary Figure 9 – Lehmann TNBC subtype tSNEs

Lehmann TNBC subtypes for cancer cell populations for TNBC patients.


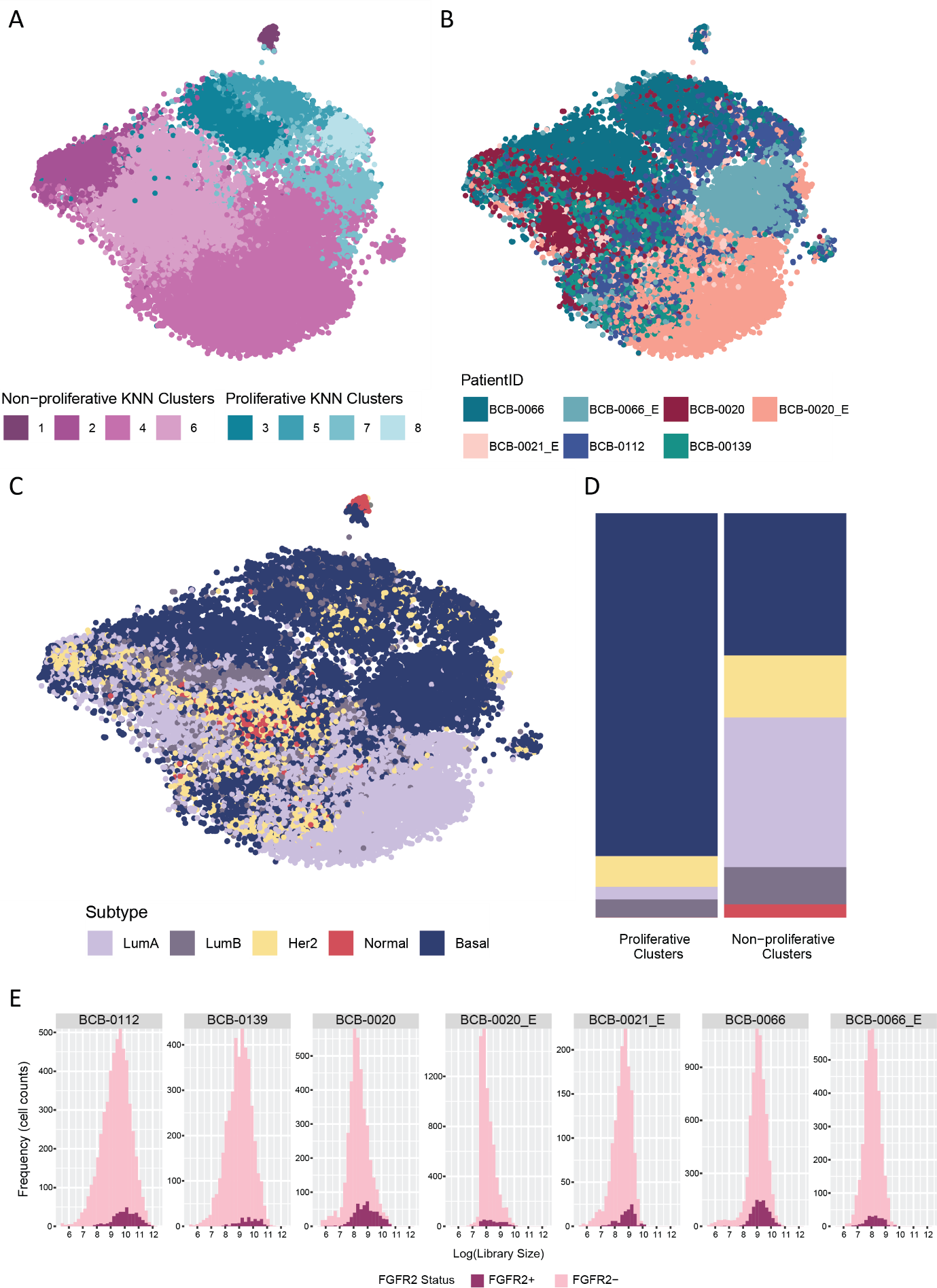


### Supplementary Figure 10 – Malignant tSNE

The tSNE of 31,628 malignant cells from Figure 3e coloured by cluster (**A**), PatientID (**B**) and predicted breast cancer subtype (**C**). The expression profiles of 7 patient samples were integrated prior to tSNE visualisation. The clusters were split based on MKI67 expression into proliferative (3, 5, 7 and 8) and non-proliferative (1, 2, 4, 6) clusters. The proportion of cells predicted as each subtype across the proliferative and non-proliferative clusters is illustrated as a proportional stacked bar chart (D). Histograms showing log-transformed library sizes for FGFR2+ and FGFR2- cells across patients (E).

# Supplementary Methods – Analysis of scRNA-seq data

## scRNA-seq data processing and quality control

Raw sequencing data were de-multiplexed and aligned to the GRCh38 genome using Cell Ranger v3.0.2 (10X Genomics). Viable cells were distinguished from empty droplets and cells with small library sizes were filtered based on outlier detection implemented in scater ^1^. Cells were also filtered if less than 200 genes were detected, or if a high percentage of reads mapped to mitochondrial genes (ranging from 25-40% depending on sample quality). Gene counts were processed using R packages scater and scran ^2^, which included log-normalisation, TPM calculation, doublet detection and dimension reduction, following standard workflows ^2^.

## Cell type annotation

A total of 5 reference datasets were applied, including two microarray references provided by SingleR ^3,4^ and 3 external single-cell datasets ^5–7^. Duplicated gene identifiers were dropped, and the AnnotationHub R package ^8^ was used to convert gene identifiers to HGNC where required.

SingleR was used to separately train each reference dataset once, deriving a list of features for each cell type in each reference. Then five classifications were obtained for each individual cell allowing investigation into the consensus of cell type across the datasets (Supplementary Table1). Cells were annotated as either malignant, mesothelial, B, T, NK or myeloid cell if they were classified into at least two of the cell type annotations listed in Table S1.

Some cells were being classified into multiple unrelated categories, for example both epithelial and T cell. Due to the sparsity of single-cell data, positive marker gene expression could not be used to confidently identify cell types and negative selection was used to distinguish some cell types. For example, EpCAM was used to negatively select lymphoid and mesothelial cells. Since EpCAM expression is not expected in the pleura, we required that B, T, NK and mesothelial cells were EpCAM negative. The small number of cells that overlapped categories were resolved manually based on their clustering location and tendency to be classified into specific labels. For example, a cell classified as both an NK and a T cell was assigned as an NK cell. Negative selection of CD3 was also used to aid the identification of NK cells.

## Immune cell annotation refinement

Myeloid cells were additionally defined as either dendritic cells or macrophages if they were classified into at least 2 of the additional labels listed in Table S1. To support the distinction of DC cells from the myeloid population, CD14, CD56 (NCAM1) and CD16 (FCGR3A) were used for the negative selection of DCs.

## Non-immune cell annotation refinement

Malignant or mesothelial cells were retained for downstream analyses of non-immune cells (Figures 3 & 4) if they were:

- Not annotated as an immune cell type in any of the ovarian cancer, LUAD or HPCA reference datasets
- Either EpCAM (malignant) or Desmin (mesothelial) positive
- If negative for the above marker, express at least two relevant markers

Epithelial markers used for cell type refinement include CLDN4, IMP3, MUC1, CDH1, CEACAM5, SCGB2A2, CLDN3 and CLDN7. Mesothelial markers used include CALB2, WT1, UPK3B, CDH2, COL1A2, S100A4 and MSLN.

## Inference of CNVs to support the identification of malignant cells

Genome-wide relative copy number estimates were calculated using the R package inferCNV ^9^ and a genomic position file was generated using the python script provided by the authors. A benign breast epithelial single-cell data was downloaded from GEO (accession code GSE113197) ^10^ to provide a non-malignant baseline for breast cancer cells. A random sample of 500 cells was taken from each individual (Ind4, Ind5, Ind6 and Ind7) and used as a reference alongside immune cells from each of our MPE patients. Raw counts from mesothelial and malignant cells were used as input, and clustering of these groups was used. The minimum average read counts per gene across reference cells were given a cut-off of 0.1 and data scaling was applied. This analysis was performed for all cells from each patient individually using the same random subset of benign breast epithelial cells as a non-malignant reference, alongside the patient’s own immune cells. For the heatmap in Figure 3d immune, mesothelial, and malignant cells were sub-sampled from each of the patients, evenly from each patient’s KNN clusters. To sample from the whole range of phenotypes present within patients, we took 25% of cells from each malignant cell cluster and 20% from each of the other clusters, hence providing more cells if a patient has more clusters.

## Breast cancer subtype classification of pseudo-bulked clusters

Reads from malignant and mesothelial cells were aggregated across cells in K-nearest neighbour (KNN) clusters into pseudo-bulk samples using scater ^1^, enabling the application of breast cancer subtype classifiers designed for bulk-sequencing data. Clusters were identified by applying the walktrap method to shared-nearest neighbours (SNN) graphs, built for each patient using k=30. Scater was used to identify outlier pseudobulk samples with small library sizes and discard them. The pseudo-bulk samples were filtered and normalised using edgeR ^11^, where genes needed a logCPM of at least 0.1 in at least 10% of samples. The Absolute Intrinsic Molecular Subtyping (AIMS) classifier ^12^, was then used to assign log-transformed pseudobulk samples to breast cancer subtypes.

Malignant cells from TNBC patients were classified into molecular subtypes using genes defined by Lehmann et al. ^13^ as gene sets. Each cell was scored for each label using VISION ^14^, as described below. The label with the highest score for each cell was taken as the molecular subtype of the cell.

## Pseudobulk differential expression analysis

To obtain pseudo-bulk samples for comparison of malignant and mesothelial populations, cells were aggregated by cell type within each patient. Only patients with sufficient populations of both mesothelial and malignant cells were used, giving eight pseudobulked samples across four patients. Pseudo-bulked samples were filtered and normalised using edgeR ^11^, where genes needed a logCPM of at least 0.5 in at least 10% of samples and patient batch effects were estimated using limma ^15^. A quasi-likelihood framework was applied to identify genes that were differentially expressed between mesothelial and malignant cells, using P-value and FDR thresholds of 0.05 and Benjamani-Hochberg P-value correction. Gene set enrichment analyses were also performed on these pseudobulk samples using limma for enrichment of GO^16^, KEGG^17^ and the Hallmark gene sets from MSigDB^18^.

For differential expression analyses between malignant cell clusters cells were aggregated by integrated cell cluster within each patient using the same workflow as above. Clusters were obtained by performing KNN clustering (K=50) on corrected data that wee integrated by patient using Seurat.

## Gene set scoring of individual cells

VISION ^14^ was used to obtain gene set scores for a CAF gene signature ^19^, various curated FGFR2-related gene sets from the Molecular Signatures Database (MSigDB), epithelial and mesenchymal gene signatures ^20^ and TNBC subtype gene sets ^13^.Counts were normalised using scater but not log-transformed, and pooling within VISION was set to false. FGFR2-related gene sets from MSigDB included PID FGF Pathway, Reactome Signaling by FGFR and Reactome Signaling by FGFR2 in disease. The FGFR2 gene was excluded from these gene sets before scoring malignant cells.

## Ligand-receptor network analysis

Ligand-receptor pairs were downloaded from CellPhoneDB ^21^, where proteins annotated as secreted were taken as ligands. The Python (v3.7.8) modules networkx and pandas was used to build a directed graph capturing ligand-receptor relationships, where ligand-to-receptor edges connected gene-level nodes. Components of complexes were represented as individual nodes, and interactions with a complex were retained for all nodes belonging to the complex.

Cell-type specific expression data was incorporated into the network, whereby ligand nodes include information about mesothelial cell populations and receptor nodes take data from malignant cell populations. For a ligand or receptor node (gene *i*), we calculate the mean log-normalised expression (E_i_) for the respective cell type as well as the proportion of the cell population that gene_i_ is expressed in (P_i_). These values are then ranked separately for mesothelial (LE_i_, LP_i_) and malignant (RE_i_, RP_i_) cell types. To overlay this expression data, edges were weighted and ranked according to the following.

LE * RE * log2(LP) * log2(RP)

The top 100 edges with the smallest product of ranks were further investigated and visualised.

References

1. McCarthy, D. J., Campbell, K. R., Lun, A. T. L. & Wills, Q. F. Scater: pre-processing, quality control, normalization and visualization of single-cell RNA-seq data in R | Bioinformatics | Oxford Academic. *Bioinformatics* **33**, 1179–1186 (2017).

2. Lun, A. T. L., McCarthy, D. J. & Marioni, J. C. A step-by-step workflow for low-level analysis of single-cell RNA-seq data [version 1; referees: 5 approved with reservations]. *F1000Research* **5**, (2016).

3. Mabbott, N. A., Baillie, J. K., Brown, H., Freeman, T. C. & Hume, D. A. An expression atlas of human primary cells: Inference of gene function from coexpression networks. *BMC Genomics* **14**, 632 (2013).

4. Schmiedel, B. J. *et al.* Impact of Genetic Polymorphisms on Human Immune Cell Gene Expression. *Cell* **175**, 1701-1715.e16 (2018).

5. Izar, B. *et al.* A single-cell landscape of high-grade serous ovarian cancer. *Nat. Med.* **26**, 1271–1279 (2020).

6. Kim, N. *et al.* Single-cell RNA sequencing demonstrates the molecular and cellular reprogramming of metastatic lung adenocarcinoma. *Nat. Commun.* **11**, (2020).

7. Adams, T. S. *et al.* Single-cell RNA-seq reveals ectopic and aberrant lung-resident cell populations in idiopathic pulmonary fibrosis. *Sci. Adv.* **6**, eaba1983 (2020).

8. Morgan, M. & Shepherd, L. AnnotationHub: Client to access AnnotationHub resources. (2020).

9. Tickle, T., Tirosh, I., Georgescu, C., Brown, M. & Haas, B. inferCNV of the Trinity CTAT Project.

10. Nguyen, Q. H. *et al.* Profiling human breast epithelial cells using single cell RNA sequencing identifies cell diversity. *Nat. Commun.* **9**, (2018).

11. Robinson, M. D., McCarthy, D. J. & Smyth, G. K. edgeR: A Bioconductor package for differential expression analysis of digital gene expression data. *Bioinformatics* **26**, 139–140 (2009).

12. Paquet, E. R. & Hallett, M. T. Absolute assignment of breast cancer intrinsic molecular subtype. *J. Natl. Cancer Inst.* **107**, (2015).

13. Lehmann, B. D. *et al.* Refinement of triple-negative breast cancer molecular subtypes: Implications for neoadjuvant chemotherapy selection. *PLoS One* **11**, (2016).

14. DeTomaso, D. *et al.* Functional interpretation of single cell similarity maps. *Nat. Commun.* **10**, 1–11 (2019).

15. Ritchie, M. E. *et al.* Limma powers differential expression analyses for RNA-sequencing and microarray studies. *Nucleic Acids Res.* **43**, e47 (2015).

16. Blake, J. A. *et al.* Gene Ontology Consortium: going forward. *Nucleic Acids Res.* **43**, D1049–D1056 (2015).

17. Kanehisa, M. & Goto, S. KEGG: Kyoto Encyclopedia of Genes and Genomes. *Nucleic Acids Res.* **28**, 27 (2000).

18. Liberzon, A. *et al.* The Molecular Signatures Database Hallmark Gene Set Collection. *Cell Syst.* **1**, 417–425 (2015).

19. Tirosh, I. *et al.* Dissecting the multicellular ecosystem of metastatic melanoma by single-cell RNA-seq. *Science (80-. ).* **352**, 189–196 (2016).

20. Tan, T. Z. *et al.* Epithelial‐mesenchymal transition spectrum quantification and its efficacy in deciphering survival and drug responses of cancer patients. *EMBO Mol. Med.* **6**, 1279–1293 (2014).

21. Efremova, M., Vento-Tormo, M., Teichmann, S. A. & Vento-Tormo, R. CellPhoneDB: inferring cell–cell communication from combined expression of multi-subunit ligand–receptor complexes. *Nat. Protoc.* **15**, 1484–1506 (2020).

Supplementary tables


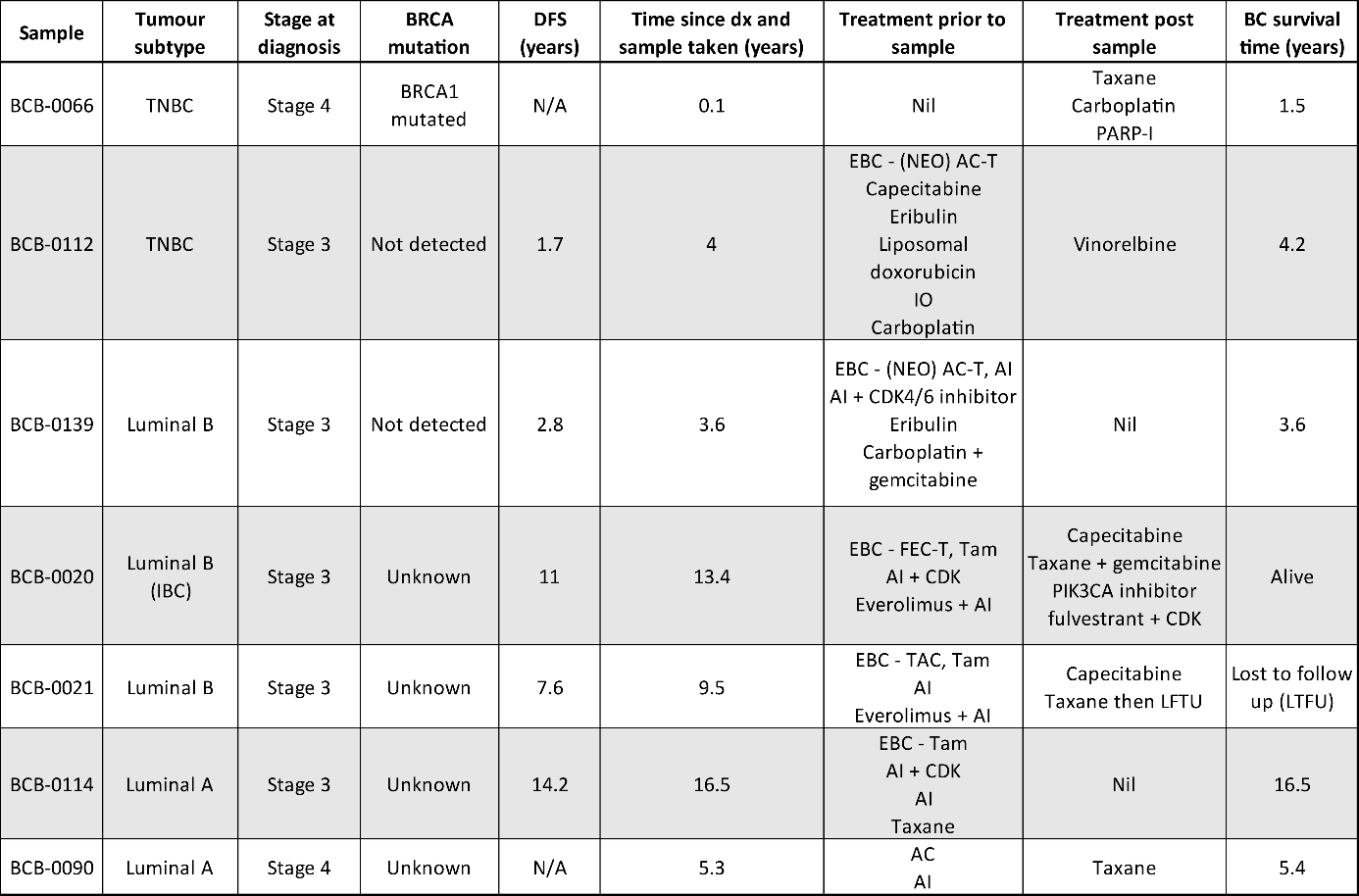


### Supplementary Table 1

Metadata and clinical information describing each patient and their treatment history. Subtypes are based on the primary tumour of the patient.

###
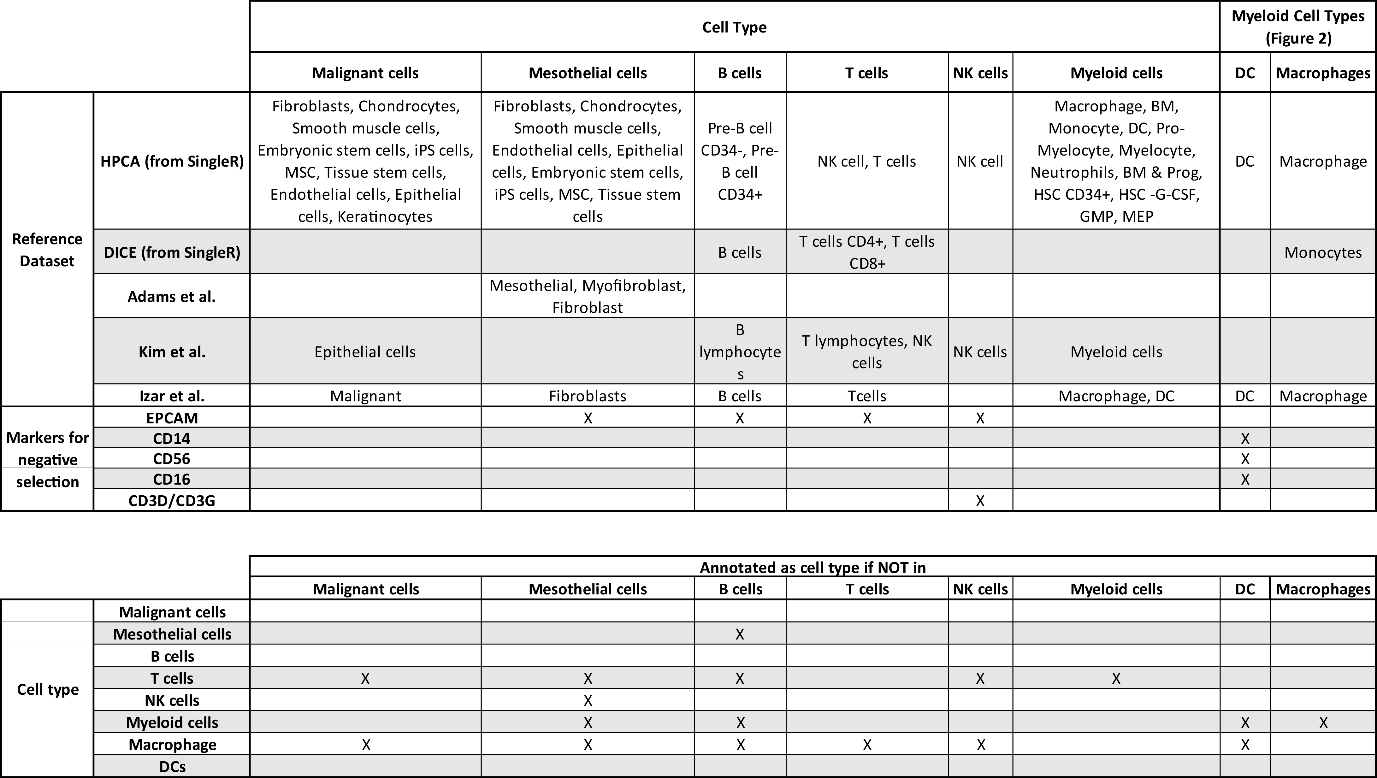


### Supplementary Table 2

Cell type annotation consensus across reference datasets. Here are the annotations used to assign a cell to each of the categories.

###
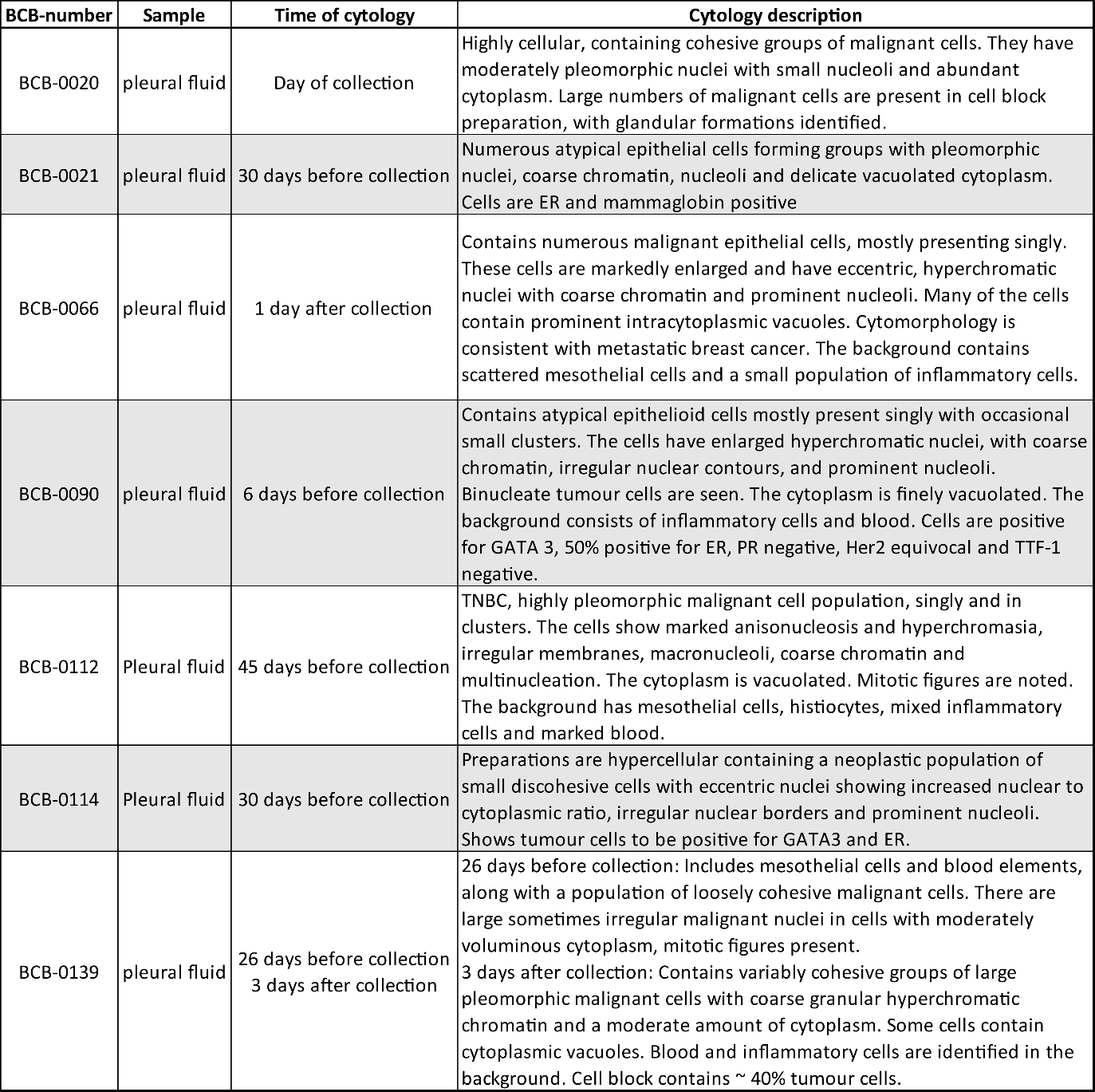


### Supplementary Table 3

Pleural effusion cytology reports containing cytology descriptions for each patient.

**Supplementary Table 4 (see file attached)**

Differentially expressed genes between mesothelial (down) and malignant (up) populations.

###

###

###

### Supplementary Table 5

Gene set enrichment analysis results for GO, KEGG and Hallmark gene sets between mesothelial (down) and malignant(up) populations.
